# Operational funds allocation through stock investment based on CODAS method and q-ROPFS aggregation operators

**DOI:** 10.1016/j.mex.2025.103640

**Published:** 2025-09-27

**Authors:** Sumbal Ali, Ikram Ullah, Salma Khan, Hasib Khan, Hisham Mohammad Alkhawar, Jehad Alzabut

**Affiliations:** a Department of Mathematics and Statistics, Hazara University, Mansehra, Khyber Pakhtunkhwa 21300, Pakistan; b School of Mathematics and Statistics, Central South University, Changsha, Hunan 410083, PR China; c Department of Mathematics and Sciences, Prince Sultan University, P.O. Box 66833, Riyadh 11586, Saudi Arabia; d Department of Mathematics, Shaheed Benazir Bhutto University, Sheringal, Dir Upper 18000, Khyber Pakhtunkhwa, Pakistan; e Department of Industrial Engineering, OSTIM Technical University, 06374 Ankara, Turkiye; f Preparatory Year Program, Computer Department, Prince Sultan University, 11586 Riyadh, Saudi Arabia

**Keywords:** q-rung orthopair picture fuzzy soft numbers, Aggregation operators, CODAS method, Decision making, Optimization

## Abstract

The q-rung orthopair picture fuzzy soft model offers a powerful framework for managing uncertainty through three membership grades: positive, neutral, and negative. In this study, we propose novel geometric aggregation operators, namely the $q-\text{ROPF}S_{t}\text{WG}$ and $q-\text{ROPF}S_{t}\text{OWG}$ operator, and establish their theoretical properties to address a key gap in existing research. These operators are applied within a MADM framework using the CODAS method, demonstrated through a real-world stock investment selection problem. To assess performance, we conduct parameter analyses, including sensitivity tests across different values of q, and comparative evaluations with existing methods. Results confirm the robustness, adaptability, and superiority of the proposed approach, offering greater flexibility and reliability in decision-making. This work bridges a significant gap by advancing the practical application of aggregation operators in dynamic real-world scenarios.

• To introduce the novel geometric aggregation operators within the context of q-rung orthopair picture fuzzy soft environment.

• The proposed aggregation operators and CODAS method are tested on a real-world multi-attribute decision-making scenario concerning stock investment selection to evaluate their effectiveness and practical applicability.

• In addition, we conduct various analysis tests with the existing methods to validate the robustness, flexibility, and superiority of the proposed model.

## Graphical abstract

**Figure:**
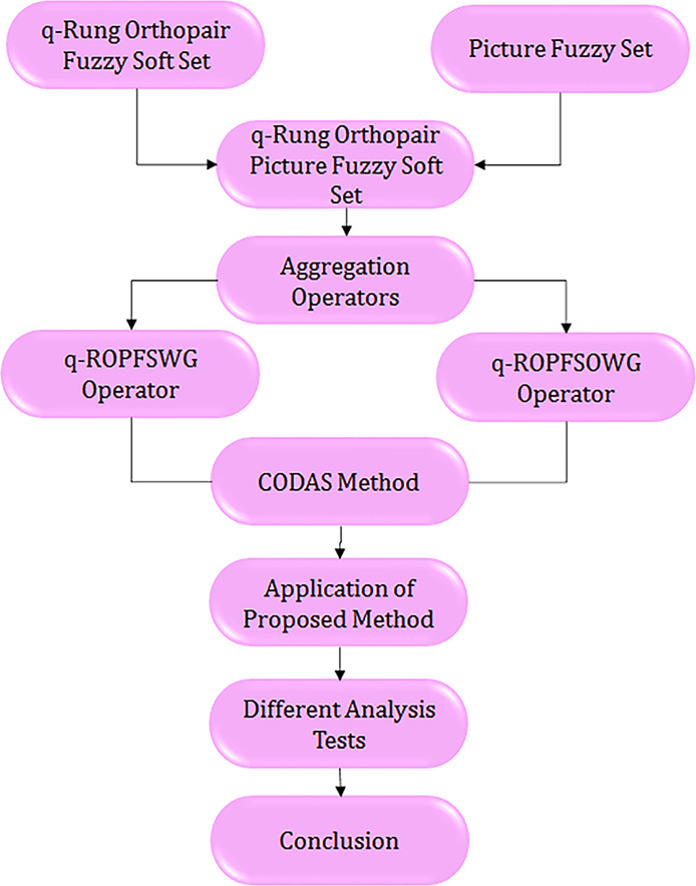

**Specifications Table**

**Table:** 

Subject area	Mathematics and Statistics
More specific subject area	Decision making and Optimization
Name of your method	CODAS Method and Aggregation Operators based on q-rung orthopair picture soft set
Name and reference of the original method	*Nill*
Resource availability	*Nill*

## Background

### Introduction

Decision-making (DM) is a primary strategy used to evaluate and select the most suitable option from the range of available alternatives. It is a difficult process because it varies significantly in various situations. Therefore, it's vital to evaluate the constraints and features of each option. The preeminent idea of a fuzzy set originated with Zadeh [1]**,** which is defined by assigning the mem-value belonging to a range of [0,1]**.** By adding a non-mem value in FS, Atanassov [2]**,** developed an intuitionistic fuzzy set which is characterized by membership degree and non-membership degree with the constraint 0 ≤ memnbership+ non-membership ≤1. For IFS, different aggregation operators were explored by many researchers. Xu [3] initiated a WA operator, while Yager [4] developed the OWA operator within the framework of IFS and the WG operator based on IFS by Xu and Yager [5]**,** using Einstein's operation, Wang and Liu [6] developed Einstein WA and Einstein OWA operators based on IFS, and Interactive aggregation operators based on IFS by He et al. [7]**,** rough aggregation operators based on IFS initiated by Chinram et al. [8] A novel approach toward generalized intuitionistic fuzzy soft sets and its applications in MADM was proposed by Khan et al. [9]**.** Some limitations are imposed on the constraint of IFS that the mem and non-mem should not exceed 1, so as to handle this type of situation. Yager [10] Introduced the Pythagorean fuzzy set with the constraint that $0\leq {\left(\text{membership}\right)}^{2}$+${\left(\text{non}-\text{membership}\right)}^{2}$≤1. So, this concept can allow experts to make optimal decisions more effectively by adding extra space to its boundary range. Many researchers explored various aggregation operators in the environment of $P_{Y}$FS. Pythagorean membership grades in multicriteria decision making were initiated by Yager [11] In 2013 also Yager & Abbasov [12] In 2013 described the relationship between Pythagorean membership grade and complex numbers was described to solve the MADM problem. Some basic result of Pythagorean fuzzy set, such as division and subtraction, and their related properties was investigated by P.eng & Yang [13] In 2015, Peng & Yuan [14] In 2016 proposed some fundamental aggregation operators such as the Pythagorean fuzzy weighted averaging operator, the Pythagorean fuzzy point operator, generalized Pythagorean fuzzy point weighted averaging operator. Using Einstein operations, Garg [15**,**^16^] proposed generalized $P_{Y}$WA and generalized $P_{Y}$WG operators were developed, and Dombi aggregation operators based on $P_{Y}$FS initiated by Khan et al. [17]**.** However, $P_{Y}$FS failed because of situation when expert assign membership = 0.85 and non-membership = 0.65, then $0\leq {\left(0.85\right)}^{2}$+${\left(0.65\right)}^{2}$≰1. So, to cope with this situation, experts need some new and generalized concepts. For this Yager [18] initiated the idea of a q-rung orthopair fuzzy set to further generalize the constraints of $P_{Y}$FS, which provided more capability and exceeded in comparison to both IFS and $P_{Y}$FS such that $0\leq {\left(\text{membership}\right)}^{qth}$+${\left(\text{non}-\text{membership}\right)}^{qth}$≤1 (q≥1). Within the context of the q-rung orthopair fuzzy set, various researchers have used different operators. Ali [19] In 2018 proposed an alternative perspective on q-rung orthopair fuzzy sets was proposed, which offers foundational insights into their structure and implications. Liu and Wang [20] initiated different AOs based on q-ROFS, showcasing relevance in MADM scenarios. Hussain et al., [21] developing group-based generalized q-rung orthopair average aggregation operators and further enhancing decision-making methodologies. Wang et al., [22] combined the concept of q-ROFS and SRS and proposed q-ROFSR sets, and their application in engineering decision-making. Liu and Liu [23] proposed Bonferroni mean operators based on q-ROFS. Xing et al., [24] initiated novel aggregation operators such as point-weighted aggregation operators, respectively, highlighting their applicability in group decision-making contexts. To address MADM challenges, Hussain et al., [25] proposed q-rung orthopair fuzzy rough set model with TOPSIS method. Liu [26] introduced an entropy-based GLDS method for selecting social capital in PPP projects, utilizing q-rung orthopair fuzzy information to enhance decision accuracy. Ye et al., [27] Proposed single variable differential calculus based on q‐rung orthopair fuzzy environment: Limit, derivative, chain rules, and their application. In frameworks like FS, IFS, $P_{Y}$FS and q-ROFS, MADM problems are addressed by mem and non-mem degrees; however, these models do not account for the neutral degree information. To bridge this gap, Coung presented a picture fuzzy set [28] which is distinguished by a triple of membership. Fuzzy set and its extension failed to handle the complex and uncertain information; the prominent study of the soft set was initiated by Molodtsov [29] by using the parameters. Different theories merged with a soft set to handle MADM issues, such as Maji [30] proposed the hybrid structure of fuzzy set and soft, and initiated fuzzy soft sets. Maji [31] initiated $\text{IF}S_{t}S$ using IFS and $S_{t}S$. Arora and Garg [32] proposed a robust aggregation operator under an intuitionistic fuzzy soft set environment, enhancing decision-making accuracy. Feng et al. [33] proposed generalized intuitionistic fuzzy soft sets and examined their use in solving MADM problems. Hayat et al. [34] proposed aggregation operator on group-based generalized intuitionistic fuzzy soft sets and application in multi-attribute decision-making methods. Hussain et al. [35] In 2020 initiated the generalized structure of q-rung orthopair fuzzy set was initiated by adding soft set, called q-rung orthopair fuzzy soft sets, and their application in the MADM problem. Zulqarnain et al. [36] proposed $P_{y}FS_{t}S$ and applied them to green supplier chain management. Joshi and Gegov [38] explored confidence levels within q-rung orthopair fuzzy aggregation operators, offering new insights for MCDM challenges. Azim et al. [37] proposed a novel concept of q-rung orthopair fuzzy rough sets and their application in solving complex MADM problems. This study expanded the existing framework of fuzzy rough sets by incorporating q-spherical fuzziness, enhancing decision-making precision. Shahzad et al. [38] explored the stability of fuzzy sets through fuzzy mappings, offering important results that methods. Ghorabaee et al. [39] proposed a new hybrid fuzzy multiple criteria decision-making approach for solving sustainable supplier selection problems.

### Research gap

The main research gap is to enhance the integration of geometric aggregation operators and the CODAS method into stock investment strategy under $q-\text{ROPF}S_{t}$ environment, ensuring these tools are robust, adaptable, and relevant in solving real-world challenges.

Our analysis of existing literature highlights the significant role of AOs in DM problems to tackle uncertainty. There are critical research gaps in the literature regarding geometric aggregation operators and the CODAS method in the context of $q-\text{ROPF}S_{t}$ environment as summarized in Table 1. So, to overcome this gap, we are merging the valuable properties of q-rung orthopair picture fuzzy soft sets with geometric aggregation operators to develop new operators such as $q-\text{ROPF}S_{t}\text{WG}$, $q-\text{ROPF}S_{t}\text{OWG}$ is capable of yielding a single choice from multiple alternatives. And also developed an algorithm based on $q-\text{ROPF}S_{t}$ Data using proposed operators and the CODAS method marks a significant advancement; however, existing research does not thoroughly investigate the performance of these algorithms across varied and dynamic environments, which provides a comprehensive analysis of evaluating their effectiveness in practical decision-making contexts. This study will contribute to a more resilient and effective decision-making framework. Some existing references are shown in Table 1.

**Table 1:** Summarize some relevant existing references.

Aggregation Operators	Description / Gaps
IFWG operator [5]	Handle uncertainty w.r.t to MD and NMD, and also no integration with soft set theory.
q-ROF$S_{t}\text{WG}$ operator [35]	Handle uncertainty w.r.t to MD and NMD with a parameterization tool, but no information about the neutral degree.
$P_{y}FS_{t}\text{WG}$ operator [36]	Handle uncertainty w.r.t to MD and NMD with a parameterization tool, but no information about the neutral degree.
q-ROF CODAS method [40]	No information regarding to neutral degree and parameterization tool
Proposed technique	Handle uncertainty w.r.t to positive MD and neutral MD, and negative MD with the parameterization tool.

### Motivations

The motivation for developing a “stock investment strategy” selection framework in the environment of *q*-rung orthopair picture fuzzy soft sets based on the CODAS method and aggregation operators needs to address several key challenges and opportunities in decision-making processes. The following points outline the primary motivations driving the proposed structure:

1. Across different disciplines, many researchers have shown keen interest in applying the concept of q-ROFS and their associated aggregation operators. Despite being widely used in many scenarios, these existing theories, like FS, IFS, $P_{Y}$FS, q-ROFS, and FFS lack of neutral-membership degree, so to address these deficiencies, we provide a more flexible and generalized structure of $q-\text{ROPF}S_{f}S$ with it constrain 0 ≤ ${\left(\alpha \right)}^{qth}+{\left(\beta \right)}^{qth}+{\left(\gamma \right)}^{qth}\leq$1 (q≥1) by extended the existing condition 0≤α≤1, 0≤α+β≤1, $0\;\leq \;{\left(\alpha \right)}^{2}+{\left(\beta \right)}^{2}\leq 1$, $0\;\leq \;{\left(\alpha \right)}^{p}+{\left(\beta \right)}^{q}\leq 1$, $0\;\leq \;{\left(\alpha \right)}^{3}+{\left(\beta \right)}^{3}\leq 1$.
2. The $q-\text{ROPF}S_{f}S$ generalize $q-\text{ROF}S_{f}S$ by offering the capability to model situations with greater generality. While $q-\text{ROF}S_{f}S$ is limited by the condition 0 ≤ ${\left(\alpha \right)}^{p}+{\left(\beta \right)}^{q}\leq$1, extended this to $0\leq {\left(\alpha \right)}^{p}+{\left(\beta \right)}^{r}+{\left(\gamma \right)}^{q}\leq 1$, incorporating a neutral degree with corresponding parameters associated with various attributes, thus enhancing the model's applicability in complex decision-making scenarios.
3. Soft sets effectively address the multi-attribute decision-making (MADM) issue through parameterization, which is not sufficiently managed by fuzzy sets and their extensions. The incorporation of soft sets into the proposed framework enhances the decision-making process by providing a structured approach to parameter management.

### Contributions

The contribution of $q-\text{ROPF}S_{f}S$ based on the CODAS method and aggregation operators need to address several key challenges and opportunities in decision-making processes. The following points outline the primary contribution driving the proposed structure:

1. The development of geometric aggregation operators based on $q-\text{ROPF}S_{t}S$ enhances the tools available for multi-attribute decision-making (MADM). These operators, $q-\text{ROPF}S_{t}W-\text{geometric}$ and $q-\text{ROPF}S_{t}\text{OW}-\text{geometric}$ extends the capabilities of decision-makers to synthesize multiple criteria and attributes effectively, which is critical for formulating an advanced stock investment strategy.
2. A detailed mathematical model and a step-by-step algorithm have been developed using aggregation operators and the CODAS method to address MADM problems using $q-\text{ROPF}S_{t}S$. This algorithm provides a systematic approach to evaluating and selecting optimal options, enhancing the practicality and applicability of the proposed model in real-world scenarios.
3. Sensitivity analysis tests have been performed to assess the reliability and influence of various parameter values “q” using the proposed aggregation operators. This analysis provides insights into the robustness of the decision-making framework and its adaptability to different scenarios and conditions, which is crucial for ensuring the reliability of stock investment strategy selection under varying circumstances.
4. The authenticity of the formulated framework has been demonstrated through rigorous comparative analysis of proposed aggregation operators with existing operators.
5. To show the superiority of the proposed model, we performed characteristic analysis with existing methods, which provides a more robust and realistic decision-making foundation compared to traditional approaches.

**Fig 1:**
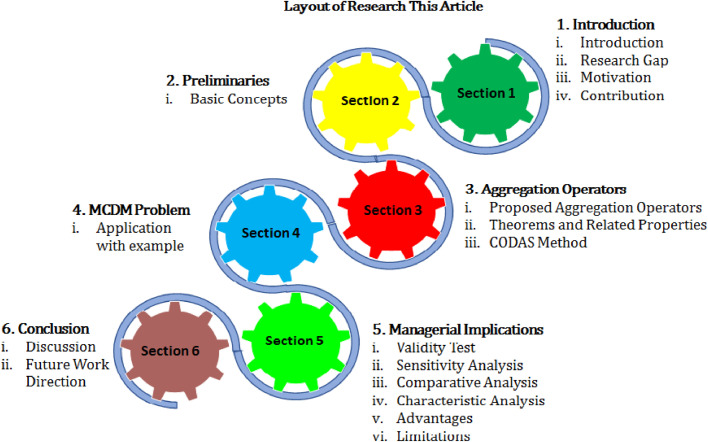
Represent the layout of the proposed work.

The remaining sections of the paper are structured and represented in Fig. 1, and also, the list of abbreviations and notation used in this research article of represented by Table 2, Table 3.

**Table 2:** Represent the list of abbreviations.

MD	Membership degree
NMD	non-membership degree
$q-\text{ROPF}S_{f}S$	q-rung orthopair Picture fuzzy soft set
AO	Averaging operator
MADM	Multi-attribute decision making
q-ROPF$S_{t}N$	q-rung orthopair picture fuzzy soft number
$q-\text{ROPF}S_{f}\text{WG}$	q-rung orthopair picture fuzzy soft weighted geometric
$q-\text{ROPF}S_{f}\text{OWG}$	q-rung orthopair picture fuzzy soft order weighted geometric
CODAS method	Combinative Distance-Based Assessment method
$S_{t}$S	Soft set

**Table 3:** Represent the list of notations.

Symbols	Meaning / Definition
U	Universal set
$N_{T}\left(u\right)$	Positive membership grade
$M_{T}\left(u\right)$	Neutral membership grade
$L_{T}\left(u\right)$	Negative membership grade
$T:₵\to q-\text{ROPF}S^{\left(\;U\;\right)}$	Mapping of q-rung orthopair picture fuzzy soft set
Q	Parameter in q-rung orthopair picture fuzzy soft set, q≥1
S$\left(T_{{Ϧ}_{\text{ij}}}\right)$	Score function of $q-\text{ROPF}S_{f}N_{s}$
$Acc(T_{{Ϧ}_{\text{ij}}})$	Accuracy function of $q-\text{ROPF}S_{f}N_{s}$
Constraint	$0\leq {\left(N_{j}\left(u_{i}\right)\right)}^{qth}+{\left(M_{j}\left(u_{i}\right)\right)}^{qth}+{\left(L_{j}\left(u_{i}\right)\right)}^{qth}\leq 1$
$\sum_{ɨ=1}^{n}\omega_{i}=1$	Weight of experts $x_{i}$
$\sum_{ʝ=1}^{m}\upsilon_{j}=1$	Weight of parameters $e_{j}$
⊗	Multiplication operational law for aggregation
⋀⋀	Minimum
∨	Maximum
$\left\{n_{1},n_{2},n_{3},n_{4},n_{5}\right\}$	Set of attributes
$\left\{L_{1},L_{2},L_{3},L_{4},L_{5}\right\}$	Set of alternatives

### Method details

#### Preliminaries

The preliminary portion provides a brief analysis of basic definitions that serve as a foundation of study, which help to establish a connection between existing theories and with proposed framework.

Definition 1 [1] A fuzzy set T can be represented mathematically as follows.

(1) $$T=\left\{u,N_{T}\left(u\right):u\epsilon U\right\}$$

with the constraint$,0\leq N_{T}\left(u\right)$≤1.

Eq. (1) illustrates the primary formulation of FS.

Definition 2 [18] A q-rung orthopair fuzzy set T in a universal set U is characterized and represented by a structure:

(2) $$T=\left\{u,N_{T}\left(u\right),M_{T}\left(u\right):u\epsilon U\right\}$$

with the constraint, $0\leq {\left(N_{T}\left(u\right)\right)}^{qth}+{\left(M_{T}\left(u\right)\right)}^{qth}\leq 1\;\left(q\geq 1\right)$.

**Fig 2:**
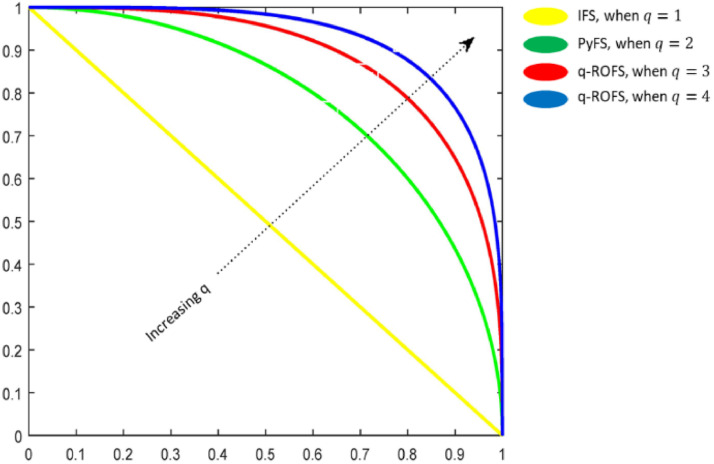
Express the assessment IFS, PFS, and q-ROF spaces.

Eq. (2) depicts the fundamental structure of q-ROFS, and Fig. 2 expresses the assessment among IFS, PFS, and q-rung orthoapair fuzzy spaces.

Definition 3 [28] A picture fuzzy set T in a universal set U is characterized and represented by a structure:

(3) $$T=\left\{u,N_{T}\left(u\right),M_{T}\left(u\right),L_{T}\left(u\right):u\epsilon U\right\}$$

with the constraint, $0\leq N_{T}\left(u\right)+M_{T}\left(u\right)+L_{T}\left(u\right)\leq 1$.

**Fig. 3:**
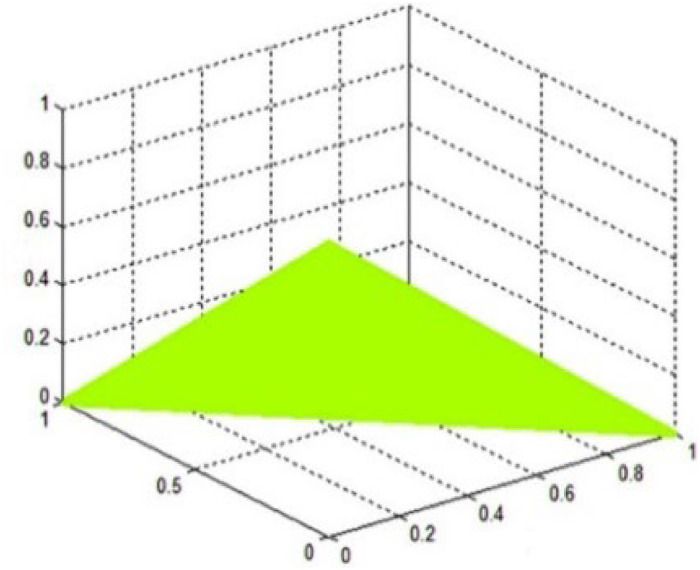
Graphical representation of picture fuzzy spaces.

Eq. (3) depicts the fundamental structure of PFS, and Fig. 3 shows the graphical representation of picture fuzzy spaces.

Definition 4 [41] A pair (T,₵) is known as $q-\text{ROPF}S_{f}S$ over the soft universe (U,₵) and ₵⊆€. Where T is given by $T:₵\to q-\text{ROPF}S^{\left(\;U\;\right)}$ and defined as

(4) $$T_{{Ϧ}_{j}}\left(u_{i}\right)=\left\{\langle u_{i},\;N_{j}\left(u_{i}\right),M_{j}\left(u_{i}\right),L_{j}\left(u_{i}\right)\rangle :u_{i}\in \;U,{Ϧ}_{j}\;\epsilon \;₵\right\}$$

With the constraint $0\leq {\left(N_{j}\left(u_{i}\right)\right)}^{qth}+{\left(M_{j}\left(u_{i}\right)\right)}^{qth}+{\left(L_{j}\left(u_{i}\right)\right)}^{qth}\leq 1\;\left(q\geq 1\right)$, and the degree of indeterminacy $\pi_{T_{{Ϧ}_{ɨʝ}}}=\sqrt[{q}]{1-({\left(N_{j}\left(u_{i}\right)\right)}^{qth}+{\left(M_{j}\left(u_{i}\right)\right)}^{qth}+{\left(L_{j}\left(u_{i}\right)\right)}^{qth})}$.

For simplicity $q-\text{ROPF}S_{f}N_{s}$ is denoted by $T_{{Ϧ}_{\text{ij}}}=\langle N_{ij},M_{ij},L_{ij}\rangle$.

**Fig. 4:**
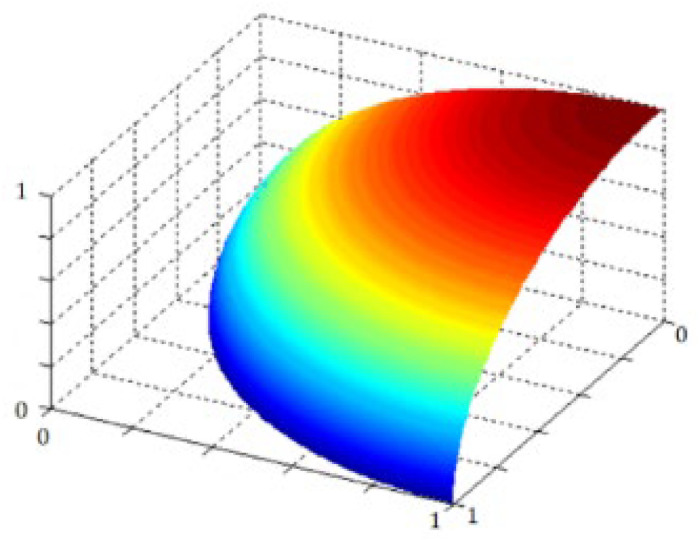
depicts the structural layout of q-ROPF$S_{f}$ spaces.

Eq. (4) expresses the fundamental structure of $q-\text{ROPF}S_{f}S$ and Fig. 4 express the visual representation of q-ROPF$S_{f}$S spaces.

Definition 5 [41] A score function and an accuracy function of $q-\text{ROPF}S_{f}N_{s}\;T_{{Ϧ}_{\text{ij}}}=\langle N_{ij},M_{ij},L_{ij}\rangle \;$can be defined as

(5) $$S\left(T_{{Ϧ}_{ij}}\right)={\left(N_{ij}\right)}^{q}-{\left(M_{ij}\right)}^{q}-{\left(L_{ij}\right)}^{q}+\left(\frac{e^{{\left(N_{ij}\right)}^{q}-{\left(M_{ij}\right)}^{q}-{\left(L_{ij}\right)}^{q}}}{e^{{\left(N_{ij}\right)}^{q}-{\left(M_{ij}\right)}^{q}-{L_{ij}}^{q}}}-\frac{1}{2}\right)\pi_{T_{{Ϧ}_{\text{ij}}}}^{q},\left(q\;\geq 1\;\right)$$

where S$\left(T_{{Ϧ}_{\text{ij}}}\right)\epsilon \left[-1,1\right]\;$an

(6) $$Acc\left(T_{{Ϧ}_{\text{ij}}}\right)={\left(N_{ij}\right)}^{q}+{\left(M_{ij}\right)}^{q}+{\left(L_{ij}\right)}^{q}$$

and $Acc\left(T_{{Ϧ}_{\text{ij}}}\right)\epsilon \left[0,1\right]$.

Let $T_{{Ϧ}_{11}}=\left(N_{11},M_{11},\;L_{11}\right)\;$and $T_{{Ϧ}_{12}}=\left(N_{12},M_{12},\;L_{12}\right)\;$be two $q-\text{ROPF}S_{f}N_{s}$. Then

I. S$\left(T_{{Ϧ}_{11}}\right)<\;S\left(T_{{Ϧ}_{12}}\right),\;T_{{Ϧ}_{11}}<T_{{Ϧ}_{12}}$
II. S$\left(T_{{Ϧ}_{11}}\right)>\;S\left(T_{{Ϧ}_{12}}\right),\;T_{{Ϧ}_{11}}>T_{{Ϧ}_{12}}$
III. S$\left(T_{{Ϧ}_{11}}\right)\;=\;S\left(T_{{Ϧ}_{12}}\right),\;$then
  a. $Acc\left(T_{{Ϧ}_{11}}\right)<\;Acc\left(T_{{Ϧ}_{12}}\right)\;\text{then}\;T_{{Ϧ}_{11}}<\;T_{{Ϧ}_{12}}$
  b. $Acc\left(T_{{Ϧ}_{11}}\right)>\;Acc\left(T_{{Ϧ}_{12}}\right)\text{then}\;T_{{Ϧ}_{11}}>\;T_{{Ϧ}_{12}}$
  c. $Acc(T_{{Ϧ}_{11}})\;$= $Acc(T_{{Ϧ}_{12}})\text{then}$ $T_{{Ϧ}_{11}}=\;T_{{Ϧ}_{12}}\;$.

### Aggregation operators

AOs are fundamental mathematical mechanisms that merge multiple input values into one representative output. These operators are essential in various fields such as decision-making, data analysis, fuzzy logic, artificial intelligence, and information fusion. The purpose of an aggregation operator is to synthesize diverse information coherently, allowing for the consolidation of individual data points, criteria, or opinions into a meaningful result.

### q-ROPF$S_{f}S$ based on weighted geometric operator

The q-rung orthopair fuzzy soft set (q-ROPF$S_{f}S$) is an extension of the traditional fuzzy set and intuitionistic fuzzy set concepts, designed to handle more uncertainty and vagueness in decision-making problems. In this context, the weighted geometric operator is used to aggregate the q-ROPF$S_{f}S$ values provide a robust method to combine different pieces of fuzzy information while accounting for the relative importance of each criterion.

Definition 6 Assume the family of $q-\text{ROPF}S_{f}N_{s}\;T_{{\ddot{e}}_{ij}}=\left(N_{ij},M_{ij},\;L_{ij}\right)\;$for experts $x_{i}$ and for parameters $e_{j}$, with $\omega_{i}$, $\upsilon_{j}\in \left[0,1\right]\;\text{such}\;\text{that}\;\sum_{i=1}^{n}\omega_{i}=1\;\text{and}\;\sum_{j=1}^{m}\upsilon_{j}=1$, which expresses the WV. Then $q-\text{ROPF}S_{f}\text{WG}$ operator is defined as $q-\text{ROPF}S_{f}\text{WG}:\;{Ɗ}^{n}\to$ Ɗ.

(7) $$q-\text{ROPF}S_{f}\text{WG}\;\;\left(T_{{\ddot{e}}_{11}},T_{{\ddot{e}}_{12}},\ldots .,T_{{\ddot{e}}_{\text{nm}}}\right)={⊗}_{ʝ=1}^{m}{\left({⊗}_{i=1}^{n}T_{{\ddot{e}}_{ij}}^{\omega_{i}}\right)}^{\upsilon_{j}}$$

Theorem 1 Let $T_{{\ddot{e}}_{ij}}=\left(N_{ij},M_{ij},\;L_{ij}\right)\;$∀ i*,j* rang from *i* to 1 an *j* to m, be the collection of $q-\text{ROPF}S_{t}N_{s}$. Then for $q-\text{ROPF}S_{f}\text{WG}$ operator: the aggregation result is defined as:

$$q-\text{ROPF}S_{f}\text{WG}\left(T_{{\ddot{e}}_{11}},T_{{\ddot{e}}_{12}},\ldots ,T_{{\ddot{e}}_{\text{nm}}}\right)={⊗}_{j=1}^{m}{\left({⊗}_{i=1}^{n}T_{{\ddot{e}}_{ij}}^{\omega_{i}}\right)}^{\upsilon_{j}}$$

(8) $$q-\text{ROPF}S_{f}\text{WG}\left(T_{{\ddot{e}}_{11}},T_{{\ddot{e}}_{12}},\ldots .,T_{{\ddot{e}}_{\text{nm}}}\right)\;=\left(\begin{array}{c} \Pi_{j=1}^{m}{\left(\Pi_{i=1}^{n}N_{ij}^{\omega_{i}}\right)}^{\upsilon_{j}},\\ {\left(1-\prod_{j=1}^{m}{\left(\prod_{i=1}^{n}{\left(1-M_{ij}^{q}\right)}^{\omega_{i}}\right)}^{\upsilon_{j}}\right)}^{\frac{1}{q}},\\ {\left(1-\prod_{j=1}^{m}{\left(\prod_{i=1}^{n}{\left(1-L_{ij}^{q}\right)}^{\omega_{i}}\right)}^{\upsilon_{j}}\right)}^{\frac{1}{q}}\; \end{array}\right)$$

where $\sum_{i=1}^{n}\omega_{i}=1\;$and $\sum_{j=1}^{m}\upsilon_{j}=1$ indicates the WV of experts and parameters.

**Proof.** For this, we will use mathematical induction. We have

$$T_{{\ddot{e}}_{11}}⊗T_{{Å}_{12}}=\left(\begin{array}{c} N_{11}N_{12},\\ \sqrt[{q}]{{\left(M_{11}\right)}^{q}+{\left(M_{12}\right)}^{q}-{\left(M_{11}\right)}^{q}{\left(M_{12}\right)}^{q}},\\ \sqrt[{q}]{{\left(L_{11}\right)}^{q}+{\left(L_{12}\right)}^{q}-{\left(L_{11}\right)}^{q}{\left(L_{12}\right)}^{q}} \end{array}\right)\text{and}$$

$$T^{\lambda }=\left({\left(Ʈ\right)}^{\lambda },\sqrt[{q}]{1-{\left(1-{\left(M\right)}^{q}\right)}^{\lambda }},\sqrt[{q}]{1-{\left(1-{\left(L\right)}^{q}\right)}^{\lambda }}\right)\text{for}\;\lambda \geq 1.$$

For n=2 and m=2, we will check.

$$q-\text{ROPF}S_{t}\text{WG}\left(T_{{\ddot{e}}_{11}},T_{{\ddot{e}}_{12}},\ldots .,T_{{\ddot{e}}_{\text{nm}}}\right)={⊗}_{j=1}^{2}{\left({⊗}_{i=1}^{2}T_{{\ddot{e}}_{ij}}^{\omega_{i}}\right)}^{\upsilon_{j}}$$

$$=\;{\left({⊗}_{i=1}^{2}{Å}_{{\ddot{e}}_{11}}^{\omega_{i}}\right)}^{\upsilon_{1}}⊗{\left({⊗}_{i=1}^{2}T_{{\ddot{e}}_{12}}^{\omega_{i}}\right)}^{\upsilon_{2}}$$

$$=\;{\left(T_{{\ddot{e}}_{11}}^{\omega_{1}}⊗T_{{\ddot{e}}_{21}}^{\omega_{2}}\right)}^{\upsilon_{1}}⊗{\left(T_{{\ddot{e}}_{12}}^{\omega_{1}}⊗T_{{\ddot{e}}_{22}}^{\omega_{2}}\right)}^{\upsilon_{2}}$$

We know that $T_{{\ddot{e}}_{11}}^{\omega_{1}}$ =$\left(\begin{array}{c} {\left(N_{11}\right)}^{\omega_{1}},\\ \sqrt[{q}]{1-{\left(1-{\left(M_{11}\right)}^{q}\right)}^{\omega_{1}}},\\ \sqrt[{q}]{1-{\left(1-{\left(L_{11}\right)}^{q}\right)}^{\omega_{1}}} \end{array}\right){\;\text{and}\;T}_{{\ddot{e}}_{21}}^{\omega_{2}}\;=\left(\begin{array}{c} {\left(N_{21}\right)}^{\omega_{2}},\\ \sqrt[{q}]{1-{\left(1-{\left(M_{21}\right)}^{q}\right)}^{\omega_{2}}},\\ \sqrt[{q}]{1-{\left(1-{\left(L_{21}\right)}^{q}\right)}^{\omega_{2}}} \end{array}\right)$

$$=\;{\left(T_{{\ddot{e}}_{11}}^{\omega_{1}}⊗T_{{\ddot{e}}_{21}}^{\omega_{2}}\right)}^{\upsilon_{1}}=\;{\left(\left(\begin{array}{c} {\left(N_{11}\right)}^{\omega_{1}},\\ \sqrt[{q}]{1-{\left(1-{\left(M_{11}\right)}^{q}\right)}^{\omega_{1}}},\\ \sqrt[{q}]{1-{\left(1-{\left(L_{11}\right)}^{q}\right)}^{\omega_{1}}} \end{array}\right)⊗\left(\begin{array}{c} {\left(N_{21}\right)}^{\omega_{2}},\\ \sqrt[{q}]{1-{\left(1-{\left(M_{21}\right)}^{q}\right)}^{\omega_{2}}},\\ \sqrt[{q}]{1-{\left(1-{\left(L_{21}\right)}^{q}\right)}^{\omega_{2}}} \end{array}\right)\right)}^{\upsilon_{1}}\text{and}$$

$$=\;{\left(T_{{\ddot{e}}_{12}}^{\omega_{1}}⊗T_{{\ddot{e}}_{22}}^{\omega_{2}}\right)}^{\upsilon_{2}}=\;{\left(\left(\begin{array}{c} {\left(N_{12}\right)}^{\omega_{1}},\\ \sqrt[{q}]{1-{\left(1-{\left(M_{12}\right)}^{q}\right)}^{\omega_{1}}},\\ \sqrt[{q}]{1-{\left(1-{\left(L_{12}\right)}^{q}\right)}^{\omega_{1}}} \end{array}\right)⊗\left(\begin{array}{c} {\left(N_{22}\right)}^{\omega_{2}},\\ \sqrt[{q}]{1-{\left(1-{\left(M_{22}\right)}^{q}\right)}^{\omega_{2}}},\\ \sqrt[{q}]{1-{\left(1-{\left(L_{22}\right)}^{q}\right)}^{\omega_{2}}} \end{array}\right)\right)}^{\upsilon_{2}}$$

$$=\;{\left(T_{{\ddot{e}}_{11}}^{\omega_{1}}⊗T_{{\ddot{e}}_{21}}^{\omega_{2}}\right)}^{\upsilon_{1}}⊗{\left(T_{{\ddot{e}}_{12}}^{\omega_{1}}⊗T_{{\ddot{e}}_{22}}^{\omega_{2}}\right)}^{\upsilon_{2}}$$

$$=\left({\left(\left(\begin{array}{c} {\left(N_{11}\right)}^{\omega_{1}},\\ \sqrt[{q}]{1-{\left(1-{\left(M_{11}\right)}^{q}\right)}^{\omega_{1}}},\\ \sqrt[{q}]{1-{\left(1-{\left(L_{11}\right)}^{q}\right)}^{\omega_{1}}} \end{array}\right)⊗\left(\begin{array}{c} {\left(N_{21}\right)}^{\omega_{2}},\\ \sqrt[{q}]{1-{\left(1-{\left(M_{21}\right)}^{q}\right)}^{\omega_{2}}},\\ \sqrt[{q}]{1-{\left(1-{\left(L_{21}\right)}^{q}\right)}^{\omega_{2}}} \end{array}\right)\right)}^{\upsilon_{1}}\;⊗\;{\left(\left(\begin{array}{c} {\left(N_{12}\right)}^{\omega_{1}},\\ \sqrt[{q}]{1-{\left(1-{\left(M_{12}\right)}^{q}\right)}^{\omega_{1}}},\\ \sqrt[{q}]{1-{\left(1-{\left(L_{12}\right)}^{q}\right)}^{\omega_{1}}} \end{array}\right)⊗\left(\begin{array}{c} {\left(N_{22}\right)}^{\omega_{2}},\\ \sqrt[{q}]{1-{\left(1-{\left(M_{22}\right)}^{q}\right)}^{\omega_{2}}},\\ \sqrt[{q}]{1-{\left(1-{\left(L_{22}\right)}^{q}\right)}^{\omega_{2}}} \end{array}\right)\right)}^{\upsilon_{2}}\right)$$

$$=\;{\left(\Pi_{i=1}^{2}{\left(N_{i1}\right)}^{\omega_{i}},\sqrt[{q}]{1-\Pi_{i=1}^{2}{\left(1-{\left(M_{i1}\right)}^{q}\right)}^{\omega_{i}}},\sqrt[{q}]{1-\Pi_{i=1}^{2}{\left(1-{\left(L_{i1}\right)}^{q}\right)}^{\omega_{i}}}\right)}^{\upsilon_{1}}⊗\;{\left(\Pi_{i=1}^{2}{\left(N_{i2}\right)}^{\omega_{i}},\sqrt[{q}]{1-\Pi_{i=1}^{2}{\left(1-{\left(M_{i2}\right)}^{q}\right)}^{\omega_{i}}},\sqrt[{q}]{1-\Pi_{i=1}^{2}{\left(1-{\left(L_{i2}\right)}^{q}\right)}^{\omega_{i}}}\right)}^{\upsilon_{2}}$$

$$=\;\left({\left(\Pi_{i=1}^{2}{\left(N_{i1}\right)}^{\omega_{i}}\right)}^{\upsilon_{1}},\;\sqrt[{q}]{1-{\left(\Pi_{i=1}^{2}{\left(1-{\left(M_{i1}\right)}^{q}\right)}^{\omega_{i}}\right)}^{\upsilon_{1}}},\;\sqrt[{q}]{1-{\left(\Pi_{i=1}^{2}{\left(1-{\left(L_{i1}\right)}^{q}\right)}^{\omega_{i}}\right)}^{\upsilon_{1}}}\right)⊗\left({\left(\Pi_{i=1}^{2}{\left(N_{i2}\right)}^{\omega_{i}}\right)}^{\upsilon_{2}},\;\sqrt[{q}]{1-{\left(\Pi_{i=1}^{2}{\left(1-{\left(M_{i2}\right)}^{q}\right)}^{\omega_{i}}\right)}^{\upsilon_{2}}},\;\sqrt[{q}]{1-{\left(\Pi_{i=1}^{2}{\left(1-{\left(L_{i2}\right)}^{q}\right)}^{\omega_{i}}\right)}^{\upsilon_{2}}}\right)$$

$$=\;\left(\begin{array}{c} \Pi_{j=1}^{2}{\left(\Pi_{i=1}^{2}{\left(N_{ij}\right)}^{\omega_{i}}\right)}^{\upsilon_{j}},\\ \;\sqrt[{q}]{1-\Pi_{j=1}^{2}{\left(\Pi_{i=1}^{2}{\left(1-{\left(M_{ij}\right)}^{q}\right)}^{\omega_{i}}\right)}^{\upsilon_{j}}},\;\sqrt[{q}]{1-\Pi_{j=1}^{2}{\left(\Pi_{i=1}^{2}{\left(1-{\left(L_{ij}\right)}^{q}\right)}^{\omega_{i}}\right)}^{\upsilon_{j}}} \end{array}\right)$$

The result is true for n=2 and m=2.

Secondly, we will check for n=$\kappa_{1}$ and m=$\kappa_{2}$.

$$=\;q-\text{ROPF}S_{f}\text{SWG}\left(T_{{\ddot{e}}_{11}},T_{{\ddot{e}}_{12}},\ldots .,T_{{\ddot{e}}_{\text{nm}}}\right)={⊗}_{j=1}^{\kappa_{2}}{\left({⊗}_{i=1}^{\kappa_{1}}T_{{\ddot{e}}_{ij}}^{\omega_{i}}\right)}^{\upsilon_{j}}$$

$$=\;\left(\Pi_{j=1}^{\kappa_{2}}{\left(\Pi_{i=1}^{\kappa_{1}}N_{ij}^{\omega_{i}}\right)}^{\upsilon_{j}},\sqrt[{q}]{1-\prod_{j=1}^{\kappa_{2}}{\left(\prod_{i=1}^{\kappa_{1}}{\left(1-M_{ij}^{q}\right)}^{\omega_{i}}\right)}^{\upsilon_{j}}},\;\sqrt[{q}]{1-\prod_{j=1}^{\kappa_{2}}{\left(\prod_{i=1}^{\kappa_{1}}{\left(1-L_{ij}^{q}\right)}^{\omega_{i}}\right)}^{\upsilon_{j}}}\right)$$

The result is true for n=$\kappa_{1}$ and m=$\kappa_{2}$.

Finally, for n=$\kappa_{1}$+1 and m=$\kappa_{2}+1$.

$$=\;q-\text{ROPF}S_{f}\text{WG}\left(T_{{\ddot{e}}_{11}},T_{{\ddot{e}}_{12}},\ldots .,T_{{\ddot{e}}_{\kappa_{1}\kappa_{2}}},\;T_{{\ddot{e}}_{\left(\kappa_{1}+1\right)\left(\kappa_{2}+1\right)}}\;\right)$$

$$=\;q-\text{ROPF}S_{f}\text{WG}\left(\left(T_{{\ddot{e}}_{11}},T_{{\ddot{e}}_{12}},\ldots .,T_{{\ddot{e}}_{\kappa_{1}\kappa_{2}}}\right)\times \;T_{{\ddot{e}}_{\left(\kappa_{1}+1\right)\left(\kappa_{2}+1\right)}}\right)$$

$$=\;\left\{{⊗}_{j=1}^{\kappa_{2}}{\left({⊗}_{i=1}^{\kappa_{1}}T_{{Ϧ}_{\text{ij}}}^{\omega_{i}}\right)}^{\upsilon_{j}}\right\}⊗\;{\left(T_{{Ϧ}_{\left(\kappa_{1}+1\right)\left(\kappa_{2}+1\right)}}^{\omega_{\left(\kappa_{2}+1\right)}}\right)}^{\upsilon_{\left(\kappa_{1}+1\right)}}$$

$$\left(\begin{array}{c} \Pi_{j=1}^{\kappa_{2}}{\left(\Pi_{i=1}^{\kappa_{1}}N_{ij}^{\omega_{i}}\right)}^{\upsilon_{j}},\\ \sqrt[{q}]{1-\prod_{j=1}^{\kappa_{2}}{\left(\prod_{i=1}^{\kappa_{1}}{\left(1-M_{ij}^{q}\right)}^{\omega_{i}}\right)}^{\upsilon_{j}}},\\ \;\sqrt[{q}]{1-\prod_{j=1}^{\kappa_{2}}{\left(\prod_{i=1}^{\kappa_{1}}{\left(1-L_{ij}^{q}\right)}^{\omega_{i}}\right)}^{\upsilon_{j}}} \end{array}\right)⊗{\left(T_{{Ϧ}_{\left(\kappa_{1}+1\right)\left(\kappa_{2}+1\right)}}^{\omega_{\left(\kappa_{2}+1\right)}}\right)}^{\upsilon_{\left(\kappa_{1}+1\right)}}$$

$$=\;\left(\begin{array}{c} \Pi_{j=1}^{\left(\kappa_{2}+1\right)}{\left(\Pi_{i=1}^{\left(\kappa_{1}+1\right)}N_{ij}^{\omega_{i}}\right)}^{\upsilon_{j}},\\ \sqrt[{q}]{1-\prod_{j=1}^{\left(\kappa_{2}+1\right)}{\left(\prod_{i=1}^{\left(\kappa_{1}+1\right)}{\left(1-M_{ij}^{q}\right)}^{\omega_{i}}\right)}^{\upsilon_{j}}},\;\\ \sqrt[{q}]{1-\prod_{j=1}^{\left(\kappa_{2}+1\right)}{\left(\prod_{i=1}^{\left(\kappa_{1}+1\right)}{\left(1-L_{ij}^{q}\right)}^{\omega_{i}}\right)}^{\upsilon_{j}}} \end{array}\right)$$

Hence, the result is true for n=$\kappa_{1}$+1 and m=$\kappa_{2}+1$. So, for all values of m and n that are greater than or equal to 1, the mathematical induction is true. For the collection of any $q-\text{ROPF}S_{t}N_{s}$ $T_{{\ddot{e}}_{\text{ij}}}$=$\left(N_{\text{ij}},M_{\text{ij}},\;L_{\text{ij}}\right)$ where 0 ≤ $N_{ij},M_{ij},L_{ij}\leq 1,\;s\text{atisify}\;\text{the}\;\text{condition}\;0\;\leq \;N_{ij}^{q}+M_{ij}^{q}+L_{ij}^{q}\leq 1\;$for expert x_i_ and parameters e_j_, with the WV $\omega_{i}$ such that $\sum_{i=1}^{n}\omega_{i}=1\;$and $\upsilon_{j}\;$such that $\sum_{j=1}^{m}\upsilon_{j}=1$. So,

$$\Rightarrow \;0\;\leq \;N_{ij}\leq 1$$

$$\Rightarrow \;0\;\leq \Pi_{i=1}^{n}\;N_{ij}^{\omega_{i}}\leq 1$$

$$\Rightarrow \;0\;\leq {\;\Pi }_{j=1}^{m}{\left(\Pi_{i=1}^{n}N_{ij}^{\omega_{i}}\right)}^{\upsilon_{j}}\leq 1.$$

Next,

$$0\;\leq \;M_{ij}\leq 1\;\Rightarrow \;\;0\;\leq 1-M_{ij}\leq 1\;\Rightarrow \;0\;\leq \;{\left(1-M_{ij}^{q}\right)}^{\omega_{i}}\leq 1\;\Rightarrow \;0\;\leq \;\Pi_{i=1}^{n}{\left(1-M_{ij}^{q}\right)}^{\omega_{i}}\leq 1$$

$$\Rightarrow \;0\;\leq \;\Pi_{j=1}^{m}{\left(\Pi_{i=1}^{n}{\left(1-M_{ij}^{q}\right)}^{\omega_{i}}\right)}^{\upsilon_{j}}\leq 1\;\Rightarrow \;0\;\leq \;\sqrt[{q}]{\Pi_{j=1}^{m}{\left(\Pi_{i=1}^{n}{\left(1-M_{ij}^{q}\right)}^{\omega_{i}}\right)}^{j}}\leq 1$$

and similarly, for

$$0\;\leq L_{ij}\leq 1\;\Rightarrow \;0\;\leq 1-L_{\text{ij}}\leq 1\;\Rightarrow \;0\;\leq \;{\left(1-L_{ij}^{q}\right)}^{\omega_{i}}\leq 1\;\Rightarrow \;0\;\leq \;\Pi_{i=1}^{n}{\left(1-L_{ij}^{q}\right)}^{\omega_{i}}\leq$$

$$\Rightarrow \;0\;\leq \;\Pi_{j=1}^{m}{\left(\Pi_{i=1}^{n}{\left(1-L_{ij}^{q}\right)}^{\omega_{i}}\right)}^{\upsilon_{j}}\leq 1$$

$\Rightarrow \;0\;\leq \;\sqrt[{q}]{\Pi_{j=1}^{m}{\left(\Pi_{i=1}^{n}{\left(1-L_{ij}^{q}\right)}^{\omega_{i}}\right)}^{j}}\leq 1$.

As we know that $0\leq {\;Ʈ}_{ij}^{q}$+$\;M_{ij}^{q}+\;L_{ij}^{q}\leq 1$

$$\Rightarrow \;\;N_{ij}^{q}\leq 1+\left(1-\;M_{ij}^{q},1-\;L_{ij}^{q}\right)$$

$$\Rightarrow \;\;N_{ij}^{q}\leq 1-\;M_{ij}^{q}+1-\;L_{ij}^{q}$$

$$\Rightarrow \;\Pi_{i=1}^{n}{\left(\;N_{ij}^{q}\right)}^{\omega_{i}}\leq \Pi_{i=1}^{n}{\left(1-M{ƚ}_{ij}^{q}\right)}^{\omega_{i}}+\Pi_{i=1}^{n}{\left(1-\;L_{ij}^{q}\right)}^{\omega_{i}}$$

$$\Rightarrow \;\Pi_{j=1}^{m}{\left(\Pi_{i=1}^{n}{\left(\;N_{ij}^{q}\right)}^{\omega_{i}}\right)}^{\upsilon_{j}}\leq \Pi_{j=1}^{m}{\left(\Pi_{i=1}^{n}{\left(1-\;M_{ij}^{q}\right)}^{\omega_{i}}\right)}^{\upsilon_{j}}+\Pi_{j=1}^{m}{\left(\Pi_{i=1}^{n}{\left(1-\;L_{ij}^{q}\right)}^{\omega_{i}}\right)}^{\upsilon_{j}}$$

$$0\;\leq \;{\left\{\Pi_{j=1}^{m}{\left(\Pi_{i=1}^{n}{\left(\;N_{ij}^{q}\right)}^{\omega_{i}}\right)}^{\upsilon_{j}}\right\}}^{q}+{\left\{\sqrt[{q}]{1-\prod_{j=1}^{m}{\left(\prod_{i=1}^{n}{\left(1-M_{ij}^{q}\right)}^{\omega_{i}}\right)}^{\upsilon_{j}}}\right\}}^{q}+{\left\{\sqrt[{q}]{1-\prod_{j=1}^{m}{\left(\prod_{i=1}^{n}{\left(1-L_{ij}^{q}\right)}^{\omega_{i}}\right)}^{\upsilon_{j}}}\right\}}^{q}$$

by Equation (8)

$$0\leq 1-\left(\prod_{j=1}^{m}{\left(\prod_{i=1}^{n}{\left(1-M_{ij}^{q}\right)}^{\omega_{i}}\right)}^{\upsilon_{j}}+\Pi_{j=1}^{m}{\left(\Pi_{i=1}^{n}{\left(1-\;L_{ij}^{q}\right)}^{\omega_{i}}\right)}^{\upsilon_{j}}\right)+\left(\prod_{j=1}^{m}{\left(\prod_{i=1}^{n}{\left(1-M_{ij}^{q}\right)}^{\omega_{i}}\right)}^{\upsilon_{j}}+\Pi_{j=1}^{m}{\left(\Pi_{i=1}^{n}{\left(1-\;L_{ij}^{q}\right)}^{\omega_{i}}\right)}^{\upsilon_{j}}\right)=1$$

Therefore,

$$0\leq {\left\{\Pi_{j=1}^{m}{\left(\Pi_{i=1}^{n}N_{ij}^{\omega_{i}}\right)}^{\upsilon_{j}}\right\}}^{q}+$$

$${\left\{\sqrt[{q}]{1-\prod_{j=1}^{m}{\left(\prod_{i=1}^{n}{\left(1-M_{ij}^{q}\right)}^{\omega_{i}}\right)}^{\upsilon_{j}}}\right\}}^{q}+{\left\{\sqrt[{q}]{1-\prod_{j=1}^{m}{\left(\prod_{i=1}^{n}{\left(1-L_{ij}^{q}\right)}^{\omega_{i}}\right)}^{\upsilon_{j}}}\right\}}^{q}\leq 1.$$

The required result proved.

Example 1 A graduate student wants to select a high-efficiency laptop for research, data analysis, and online education. To make this decision, five different laptop modes are evaluated on the basis of their criteria, and these criteria are designed to reflect the performance and capabilities of each device.

Set of Alternatives (Laptop Models):

$$A\;=\;\left\{a_{1},\;a_{2},\;a_{3},a_{4},a_{5}\right\}$$

$a_{1}=\;$Dell, $a_{2}$= HP, $a_{3}=\;$MacBook, $a_{4}$= ASUS ZenBook and $a_{5}$= Lenovo

Set of criteria:

C= {$c_{1}$, $c_{2}$, $c_{3},c_{4},c_{5}$}

$c_{1}\;$= Speed, $c_{2\;}$= RAM, $c_{3\;}$= Battery time, $c_{4}\;$= Display and $c_{5}$= SSD Storage.

Weight Vector:

Weight of Experts ${x_{i}}_{\;}\;$ω = ${\left(0.16,0.26,0.15,0.20,0.23\right)}^{T}$

Weight of Criteria $e_{j}\;$υ=${\left(0.28,0.20,0.1,0.15,0.27\right)}^{T}$

Table 4. represents the assessment of each alternative concerning its parameters in the form of $q-\text{ROPF}S_{t}N_{s}$ $\left(N_{ij},M_{ij},\;L_{ij}\right)$, where $N_{ij},M_{ij}\;\text{and}\;L_{ij}$, represent the degree of positive, neutral and negative membership function.

**Table 4:** $q-\text{ROPF}S_{t}N_{s}$$T_{{\ddot{e}}_{ij}}$= $(N_{ij},M_{ij},\;L_{ij})\;$for q ≥ 1.

Alternatives	$c_{1}$	$c_{2}$	$c_{3}$	$c_{4}$	$c_{5}$
$a_{1}$	(.3, .4, .2)	(.3, .3, .4)	(.2, .2, .1)	(.8, .1, .1)	(.5, .3, .1)
$a_{2}$	(.1, .1, .4)	(.2, .1, .3)	(.3, .4, .3)	(.4, .2, .4)	(.4, .3, .2)
$a_{3}$	(.7, .1, .1)	(.5, .4, .1)	(.4, .1, .2)	(.1, .2, .1)	(.6, .2, .1)
$a_{4}$	(.5, .3, .2)	(.4, .3, .2)	(.1, .3, .3)	(.5, .1, .4)	(.3, .2, .3)
$a_{5}$	(.4, .2, .1)	(.7, .1, .2)	(.5, .4, .1)	(.3, .2, .1)	(.2, .4, .1)

Step-by-Step computation using $q-\text{ROPF}S_{t}\text{WG}$ operator, we have

$$q-\text{ROPF}S_{t}\text{WG}\left(T_{{\ddot{e}}_{11}},T_{{\ddot{e}}_{12}},\ldots .,T_{{\ddot{e}}_{\text{nm}}}\right)\;=\;\left(\begin{array}{c} \Pi_{j=1}^{m}{\left(\Pi_{i=1}^{n}N_{ij}^{\omega_{i}}\right)}^{\upsilon_{j}},\sqrt[{q}]{1-\prod_{j=1}^{m}{\left(\prod_{i=1}^{n}{\left(1-M_{ij}^{q}\right)}^{\omega_{i}}\right)}^{\upsilon_{j}}}\\ ,\\ \sqrt[{q}]{1-\prod_{j=1}^{m}{\left(\prod_{i=1}^{n}{\left(1-L_{ij}^{q}\right)}^{\omega_{i}}\right)}^{\upsilon_{j}}} \end{array}\right)$$

Compute aggregated positive, neutral and negative membership values in $q-\text{ROPF}S_{t}\text{WG}$.

$$=\left(\begin{array}{c} \left(\begin{array}{c} {\left\{0.3^{0.16}0.1^{0.26}0.7^{0.15}0.5^{0.20}0.4^{0.23}\right\}}^{0.28}\\ {\left\{0.3^{0.16}0.2^{0.26}0.5^{0.15}0.4^{0.20}0.7^{0.23}\right\}}^{0.20}\\ {\left\{0.2^{0.16}0.3^{0.26}0.4^{0.15}0.1^{0.20}0.5^{0.23}\right\}}^{0.1}\\ {\left\{0.8^{0.16}0.4^{0.26}0.1^{0.15}0.5^{0.20}0.3^{0.23}\right\}}^{0.15}\\ {\left\{0.8^{0.16}0.4^{0.26}0.6^{0.15}0.3^{0.20}0.2^{0.23}\right\}}^{0.27} \end{array}\right),\\ \sqrt[{3}]{\begin{array}{c} 1-{\left\{{\left(1-0.4^{3}\right)}^{0.16}{\left(1-0.1^{3}\right)}^{0.26}{\left(1-0.1^{3}\right)}^{0.15}{\left(1-0.3^{3}\right)}^{0.20}{\left(1-0.2^{3}\right)}^{0.23}\right\}}^{0.28}\\ 1-{\left\{{\left(1-0.3^{3}\right)}^{0.16}{\left(1-0.1^{3}\right)}^{0.26}{\left(1-0.4^{3}\right)}^{0.15}{\left(1-0.3^{3}\right)}^{0.20}{\left(1-0.1^{3}\right)}^{0.23s}\right\}}^{0.20}\\ 1-{\left\{{\left(1-0.2^{3}\right)}^{0.16}{\left(1-0.4^{3}\right)}^{0.26}{\left(1-0.1^{3}\right)}^{0.15}{\left(1-0.3^{3}\right)}^{0.20}{\left(1-0.4^{3}\right)}^{0.23}\right\}}^{0.1}\\ 1-{\left\{{\left(1-0.1^{3}\right)}^{0.16}{\left(1-0.2^{3}\right)}^{0.26}{\left(1-0.2^{3}\right)}^{0.15}{\left(1-0.1^{3}\right)}^{0.20}{\left(1-0.2^{3}\right)}^{0.23}\right\}}^{0.15}\\ 1-{\left\{{\left(1-0.3^{3}\right)}^{0.16}{\left(1-0.3^{3}\right)}^{0.26}{\left(1-0.2^{3}\right)}^{0.15}{\left(1-0.2^{3}\right)}^{0.20}{\left(1-0.4^{3}\right)}^{0.23}\right\}}^{0.27} \end{array}},\\ \sqrt[{3}]{\begin{array}{c} 1-{\left\{{\left(1-0.2^{3}\right)}^{0.16}{\left(1-0.4^{3}\right)}^{0.26}{\left(1-0.1^{3}\right)}^{0.15}{\left(1-0.2^{3}\right)}^{0.20}{\left(1-0.1^{3}\right)}^{0.23}\right\}}^{0.28}\\ 1-{\left\{{\left(1-0.4^{3}\right)}^{0.16}{\left(1-0.3^{3}\right)}^{0.26}{\left(1-0.1^{3}\right)}^{0.15}{\left(1-0.2^{3}\right)}^{0.20}{\left(1-0.2^{3}\right)}^{0.23}\right\}}^{0.20}\\ 1-{\left\{{\left(1-0.1^{3}\right)}^{0.16}{\left(1-0.3^{3}\right)}^{0.26}{\left(1-0.2^{3}\right)}^{0.15}{\left(1-0.3^{3}\right)}^{0.20}{\left(1-0.1^{3}\right)}^{0.23}\right\}}^{0.1}\\ 1-{\left\{{\left(1-0.1^{3}\right)}^{0.16}{\left(1-0.4^{3}\right)}^{0.26}{\left(1-0.1^{3}\right)}^{0.15}{\left(1-0.4^{3}\right)}^{0.20}{\left(1-0.1^{3}\right)}^{0.23}\right\}}^{0.15}\\ 1-{\left\{{\left(1-0.1^{3}\right)}^{0.16}{\left(1-0.2^{3}\right)}^{0.26}{\left(1-0.1^{3}\right)}^{0.15}{\left(1-0.3^{3}\right)}^{0.20}{\left(1-0.1^{3}\right)}^{0.23}\right\}}^{0.27} \end{array}} \end{array}\right)$$

After computation the aggregated q-rung orthopair picture fuzzy soft values, we get

=(0.3334,0.2788,0.2625).

### q-ROPF$S_{f}S$ based on ordered weighted geometric operator

The q-rung orthopair fuzzy soft set (q-ROPF$S_{t}S$) is an advanced tool in fuzzy set theory designed to manage higher degrees of uncertainty compared to traditional fuzzy sets. The ordered weighted geometric (OWG) operator is a sophisticated aggregation operator that is used to combine multiple q-ROPF$S_{t}S$ values in a way that not only considers the importance of each criterion but also takes into account the relative ordering of the input values. When we combine the concepts of q-ROPF$S_{t}S$ and the ordered weighted geometric operator, specifically in the context of a transformation denoted by q-ROPF$S_{t}\text{SOWG}$ , we create a powerful tool for decision-making in environments with high uncertainty and complexity.

Definition 7 Assume the family of $q-\text{ROPF}S_{f}N_{s}\;T_{{\ddot{e}}_{ij}}=\left(N_{ij},M_{ij},\;L_{ij}\right)\;$for experts $x_{i}$ and for parameters $e_{j}$, with $\omega_{i}$, $\upsilon_{j}\in \left[0,1\right]\;\text{such}\;\text{that}\;\sum_{i=1}^{n}\omega_{i}=1\;\text{and}\;\sum_{j=1}^{m}\upsilon_{j}=1$, which expresses the WV. Then $q-\text{ROPF}S_{f}\text{OWG}$ operator is defined as $q-\text{ROPF}S_{f}\text{OWG}:\;{Ɗ}^{n}\to$ Ɗ.

(9) $$q-\text{ROPF}S_{f}\text{OWG}\left(T_{{\ddot{e}}_{11}},T_{{\ddot{e}}_{12}},\ldots .,T_{{\ddot{e}}_{\text{nm}}}\right)={⊗}_{j=1}^{m}{\left({⊗}_{i=1}^{n}T_{\sigma {\ddot{e}}_{ij}}^{\omega_{i}}\right)}^{\upsilon_{j}}$$

Theorem 2 Let $T_{{\ddot{e}}_{ij}}=\left(N_{ij},M_{ij},\;L_{ij}\right)\;$for the rang from *i* to 1 and *j* to m, be the collection of $q-\text{ROPF}S_{t}N_{s}$. Then for q-ROPF$S_{f}$OWG operator: the aggregation result is defined:

$$q-\text{ROPF}S_{f}\text{OWG}\left(T_{{\ddot{e}}_{11}},T_{{\ddot{e}}_{12}},\ldots .,T_{{\ddot{e}}_{\text{nm}}}\right)={⊗}_{j=1}^{m}{\left({⊗}_{i=1}^{n}T_{\sigma {\ddot{e}}_{ij}}^{\omega_{i}}\right)}^{\upsilon_{j}}$$

(10) $$q-\text{ROPF}S_{f}\text{OWG}\left(T_{{\ddot{e}}_{11}},T_{{\ddot{e}}_{12}},\ldots .,T_{{\ddot{e}}_{\text{nm}}}\right)=\;\left(\begin{array}{c} \Pi_{j=1}^{m}{\left(\Pi_{i=1}^{n}N_{\sigma ij}^{\omega_{ɨ}}\right)}^{\upsilon_{j}},\\ \sqrt[{q}]{1-\prod_{ʝ=1}^{m}{\left(\prod_{i=1}^{n}{\left(1-M_{\sigma ij}^{q}\right)}^{\omega_{i}}\right)}^{\upsilon_{j}}},\\ \sqrt[{q}]{1-\prod_{j=1}^{m}{\left(\prod_{i=1}^{n}{\left(1-L_{\sigma ij}^{q}\right)}^{\omega_{i}}\right)}^{\upsilon_{j}}} \end{array}\right)$$

Where $T_{\sigma {\ddot{e}}_{ij}}=\left(N_{\sigma ij},M_{\sigma ij},\;L_{\sigma ij}\right)$ represent the largest values of the collection of i×j of $q-\text{ROPF}S_{t}N_{s}$.

**Proof.** No new or complex steps are required. Theorem 1 already contains the conditions that lead directly to this result.

Example 2 By using the score function, we take the collection of $q-\text{ROPF}S_{t}N_{s}$ $T_{{\ddot{e}}_{ij}}=\left(N_{ij},M_{ij},\;L_{ij}\right)\;$From Table 3 of Example 1. This gives the transformed values shown in in “Table 5” in the form of $T_{\sigma {\ddot{e}}_{ij}}=\left(N_{\sigma ij},M_{\sigma ij},\;L_{\sigma ij}\right)$.

**Table 5:** $q-\text{ROPF}S_{t}N_{s}$$T_{\sigma {\ddot{e}}_{ij}}=\left(N_{\sigma ij},M_{\sigma ij},\;L_{\sigma ij}\right)\;$for q ≥ 1.

Alternatives	$c_{1}$	$c_{2}$	$c_{3}$	$c_{4}$	$c_{5}$
$a_{1}$	(.7, .1, .1)	(.7, .1, .2)	(.5, .4, .1)	(.8, .1, .1)	(.6, .2, .1)
$a_{2}$	(.5, .3, .2)	(.5, .4, .1)	(.4, .1, .2)	(.5, .1, .4)	(.5, .3, .1)
$a_{3}$	(.4, .2, .1)	(.4, .3, .2)	(.3, .4, .3)	(.4, .2, .4)	(.4, .3, .2)
$a_{4}$	(.3, .4, .2)	(.3, .3, .4)	(.2, .2, .1)	(.3, .2, .1)	(.3, .2, .3)
$a_{5}$	(.1, .1, .4)	(.2, .1, .3)	(.1, .3, .3)	(.1, .2, .1)	(.2, .4, .1)

Step-by-Step computation using $q-\text{ROPF}S_{t}\text{OWG}$ operator. The operator is define as

$$q-\text{ROPF}S_{f}\text{OWG}\left(T_{{\ddot{e}}_{11}},T_{{\ddot{e}}_{12}},\ldots .,T_{{\ddot{e}}_{\text{nm}}}\right)=\;\left(\begin{array}{c} \Pi_{j=1}^{m}{\left(\Pi_{i=1}^{n}N_{\sigma ij}^{\omega_{i}}\right)}^{\upsilon_{j}},\\ \sqrt[{q}]{1-\prod_{j=1}^{m}{\left(\prod_{i=1}^{n}{\left(1-M_{\sigma ij}^{q}\right)}^{\omega_{i}}\right)}^{\upsilon_{j}}},\\ \sqrt[{q}]{1-\prod_{j=1}^{m}{\left(\prod_{i=1}^{n}{\left(1-L_{\sigma ij}^{q}\right)}^{\omega_{i}}\right)}^{\upsilon_{j}}} \end{array}\right)$$

Compute aggregated positive, neutral and negative membership values in $q-\text{ROPF}S_{t}\text{OWG}$.

$$=\left(\begin{array}{c} \left(\begin{array}{c} {\left\{(0.7^{0.16}0.5^{0.26}0.4^{0.15}0.3^{0.20}0.1^{0.23}\right\}}^{0.28}\\ {\left\{0.7^{0.16}0.5^{0.26}0.4^{0.15}0.3^{0.20}0.2^{0.23}\right\}}^{0.20}\\ {\left\{0.5^{0.16}0.4^{0.26}0.3^{0.15}0.2^{0.20}0.1^{0.23}\right\}}^{0.1}\\ {\left\{0.8^{0.16}0.5^{0.26}0.4^{0.15}0.3^{0.20}0.1^{0.23}\right\}}^{0.15}\\ {\left\{0.6^{0.16}0.5^{0.26}0.4^{0.15}0.3^{0.20}0.2^{0.23}\right\}}^{0.27} \end{array}\right),\\ \sqrt[{3}]{\begin{array}{c} 1-{\left\{{\left(1-0.1\right)}^{0.16}{\left(1-0.3^{3}\right)}^{0.26}{\left(1-0.2^{3}\right)}^{0.15}{\left(1-0.4^{3}\right)}^{0.20}{\left(1-0.1^{3}\right)}^{0.23}\right\}}^{0.28}\\ 1-{\left\{{\left(1-0.1^{3}\right)}^{0.16}{\left(1-0.4^{3}\right)}^{0.26}{\left(1-0.3^{3}\right)}^{0.15}{\left(1-0.3^{3}\right)}^{0.20}{\left(1-0.1^{3}\right)}^{0.23}\right\}}^{0.20}\\ 1-{\left\{{\left(1-0.4^{3}\right)}^{0.16}{\left(1-0.1^{3}\right)}^{0.26}{\left(1-0.4^{3}\right)}^{0.15}{\left(1-0.2^{3}\right)}^{0.20}{\left(1-0.3^{3}\right)}^{0.23}\right\}}^{0.1}\\ 1-{\left\{{\left(1-0.1^{3}\right)}^{0.16}{\left(1-0.1^{3}\right)}^{0.26}{\left(1-0.2^{3}\right)}^{0.15}{\left(1-0.2^{3}\right)}^{0.20}{\left(1-0.2^{3}\right)}^{0.23}\right\}}^{0.15}\\ 1-{\left\{{\left(1-0.2^{3}\right)}^{0.16}{\left(1-0.3^{3}\right)}^{0.26}{\left(1-0.3^{3}\right)}^{0.15}{\left(1-0.2^{3}\right)}^{0.20}{\left(1-0.4^{3}\right)}^{0.23}\right\}}^{0.27} \end{array}},\\ \sqrt[{3}]{\begin{array}{c} 1-{\left\{{\left(1-0.1^{3}\right)}^{0.16}{\left(1-0.2^{3}\right)}^{0.26}{\left(1-0.1^{3}\right)}^{0.15}{\left(1-0.2^{3}\right)}^{0.20}{\left(1-0.4^{3}\right)}^{0.23}\right\}}^{0.28}\\ 1-{\left\{{\left(1-0.2^{3}\right)}^{0.16}{\left(1-0.1^{3}\right)}^{0.26}{\left(1-0.2^{3}\right)}^{0.15}{\left(1-0.4^{3}\right)}^{0.20}{\left(1-0.3^{3}\right)}^{0.23}\right\}}^{0.20}\\ 1-{\left\{{\left(1-0.1^{3}\right)}^{0.16}{\left(1-0.2^{3}\right)}^{0.26}{\left(1-0.3^{3}\right)}^{0.15}{\left(1-0.1^{3}\right)}^{0.20}{\left(1-0.3^{3}\right)}^{0.23}\right\}}^{0.1}\\ 1-{\left\{{\left(1-0.1^{3}\right)}^{0.16}{\left(1-0.4^{3}\right)}^{0.26}{\left(1-0.4^{3}\right)}^{0.15}{\left(1-0.1^{3}\right)}^{0.20}{\left(1-0.1^{3}\right)}^{0.23}\right\}}^{0.15}\\ 1-{\left\{{\left(1-0.1^{3}\right)}^{0.16}{\left(1-0.1^{3}\right)}^{0.26}{\left(1-0.2^{3}\right)}^{0.15}{\left(1-0.3^{3}\right)}^{0.20}{\left(1-0.1^{3}\right)}^{0.23}\right\}}^{0.27} \end{array}} \end{array}\right)$$

After computation the aggregated q-rung orthopair picture fuzzy soft values, we get

=(0.3338,0.2838,0.2577)

Some Basic Properties Based on Proposed Aggregation Operators

The subsequent core properties are established based on proposed aggregation mechanisms.

Property 1 **Idempotency:**

**Statement:**

1. If the collection of $q-\text{ROPF}S_{t}N_{s}$ $T_{{\ddot{e}}_{ij}}=N_{\ddot{e}}=\left(N_{ij},M_{ij},\;L_{ij}\right)$, then$\;q-\text{ROPF}S_{t}\text{WG}\;\left(T_{{\ddot{e}}_{11}},T_{{\ddot{e}}_{12}},\ldots .,T_{{\ddot{e}}_{\text{nm}}}\right)=\;N_{\ddot{e}}$_._
2. If the collection of $q-\text{ROPF}S_{t}N_{s}$ $T_{{\ddot{e}}_{ɨʝ}}=N_{\ddot{e}}=\left(N_{ij},M_{ij},\;L_{ij}\right)$,then$q-\text{ROPF}S_{t}\text{OWG}\;\left(T_{{\ddot{e}}_{11}},T_{{\ddot{e}}_{12}},\ldots .,T_{{\ddot{e}}_{\text{nm}}}\right)=\;N_{\ddot{e}}$_._

Proof 1 If the collection of $q-\text{ROPF}S_{t}N_{s}$ $T_{{\ddot{e}}_{ɨʝ}}=N_{\ddot{e}}=\left(N_{ij},M_{ij},\;L_{ij}\right)$ then

$$q-\text{ROPF}S_{t}\text{WG}\;\left(T_{{\ddot{e}}_{11}},T_{{\ddot{e}}_{12}},\ldots .,T_{{\ddot{e}}_{\text{nm}}}\right)=\;N_{\ddot{e}}.$$

As we know that $T_{{\ddot{e}}_{ij}}=N_{\ddot{e}}=\left(N,M,\;L\right)$, then we have $\sum_{I=1}^{n}\omega_{i}$

$$q-\text{ROPF}S_{f}\text{WG}\left(T_{{\ddot{e}}_{11}},T_{{\ddot{e}}_{12}},\ldots .,T_{{\ddot{e}}_{\text{nm}}}\right)\;=\left(\begin{array}{c} \Pi_{j=1}^{m}{\left(\Pi_{i=1}^{n}{\left(N_{ij}\right)}^{\omega_{i}}\right)}^{\upsilon_{j}},\\ \sqrt[{q}]{1-\prod_{j=1}^{m}{\left(\prod_{i=1}^{n}{\left(1-M_{\text{ij}}^{q}\right)}^{\omega_{i}}\right)}^{\upsilon_{j}}},\\ \sqrt[{q}]{1-\prod_{j=1}^{m}{\left(\prod_{i=1}^{n}{\left(1-L_{ij}^{q}\right)}^{\omega_{i}}\right)}^{\upsilon_{j}}} \end{array}\right)$$

$=\left(\begin{array}{c} {\left({\left(N_{ij}\right)}^{\sum_{i=1}^{n}\omega_{i}}\right)}^{\sum_{j=1}^{m}\upsilon_{j}},\\ \sqrt[{q}]{1-{\left({\left(1-M_{ij}^{q}\right)}^{\sum_{i=1}^{n}\omega_{i}}\right)}^{\sum_{j=1}^{m}\upsilon_{j}}},\\ \sqrt[{q}]{1-{\left({\left(1-L_{ij}^{q}\right)}^{\sum_{i=1}^{n}\omega_{i}}\right)}^{\sum_{j=1}^{m}\upsilon_{j}}} \end{array}\right)\;=\left(\begin{array}{c} (N_{ij}),\\ \sqrt[{q}]{1-(1-M_{ij}^{q})},\\ \sqrt[{q}]{1-(1-L_{ij}^{q})} \end{array}\right)=\;\left(N,M,\;L\right)\;=\;N_{\ddot{e}}$_._

Therefore, the statement holds if $T_{{\ddot{e}}_{ij}}=N_{\ddot{e}}=\left(N_{ij},M_{ij},\;L_{ij}\right)$ then

$$q-\text{ROPF}S_{t}\text{WG}\;\left(T_{{\ddot{e}}_{11}},T_{{\ddot{e}}_{12}},\ldots .,T_{{\ddot{e}}_{\text{nm}}}\right)=\;N_{\ddot{e}}$$

Therefore, the statement holds if $T_{{\ddot{e}}_{ij}}=N_{\ddot{e}}=\left(N_{ij},M_{ij},\;L_{ij}\right)$ then

$$q-\text{ROPF}S_{t}\text{WG}\;\left(T_{{\ddot{e}}_{11}},T_{{\ddot{e}}_{12}},\ldots .,T_{{\ddot{e}}_{\text{nm}}}\right)=\;N_{\ddot{e}}$$

Similarly, for $q-\text{ROPF}S_{t}\text{OWG}$ operator. We consider $T_{{\ddot{e}}_{ɨʝ}}=N_{\ddot{e}}$, where $N_{\ddot{e}}=\left(₩₩,₲,\;₱\right)$, then $q-\text{ROPF}S_{t}\text{OWG}\;\left(T_{{\ddot{e}}_{11}},T_{{\ddot{e}}_{12}},\ldots .,T_{{\ddot{e}}_{\text{nm}}}\right)=\;N_{\ddot{e}}$.

$$=\left(\Pi_{j=1}^{m}{\left(\Pi_{i=1}^{n}{\left(N_{\sigma ij}\right)}^{\omega_{i}}\right)}^{\upsilon_{j}},\;\sqrt[{q}]{1-\prod_{j=1}^{m}{\left(\prod_{i=1}^{n}{\left(1-M_{\sigma \text{ij}}^{q}\right)}^{\omega_{i}}\right)}^{\upsilon_{j}},}\sqrt[{q}]{1-\prod_{j=1}^{m}{\left(\prod_{i=1}^{n}{\left(1-L_{\sigma ij}^{q}\right)}^{\omega_{i}}\right)}^{\upsilon_{j}}}\right)$$

$=(\Pi_{j=1}^{m}{\left(\Pi_{i=1}^{n}{₩₩}^{\omega_{i}}\right)}^{\upsilon_{j}},\;\sqrt[{q}]{1-\prod_{j=1}^{m}{\left(\prod_{i=1}^{n}{\left(1-{₲}^{q}\right)}^{\omega_{i}}\right)}^{\upsilon_{j}},}\sqrt[{q}]{1-\prod_{j=1}^{m}{\left(\prod_{i=1}^{n}{\left(1-{₱}^{q}\right)}^{\omega_{i}}\right)}^{\upsilon_{j}}})$

$=({\left({₩₩}^{\sum_{i=1}^{n}\omega_{i}}\right)}^{\sum_{j=1}^{m}\upsilon_{j}},\;\sqrt[{q}]{1-{\left({\left(1-{₲}^{q}\right)}^{\sum_{i=1}^{n}\omega_{i}}\right)}^{\sum_{j=1}^{m}\upsilon_{j}}},\sqrt[{q}]{1-{\left({\left(1-{₱}^{q}\right)}^{\sum_{i=1}^{n}\omega_{i}}\right)}^{\sum_{j=1}^{m}\upsilon_{j}}})$

$$=\left(₩₩,\;\sqrt[{q}]{1-\left(1-{₲}^{q}\right)},\sqrt[{q}]{1-\left(1-{₱}^{q}\right)}\right)$$

$$\left(₩₩,₲,\;₱\right)=\;N_{\ddot{e}}$$

Hence the result is hold for $q-\text{ROPF}S_{t}\text{OWG}$ operator.

Property 2 **Bondedness**:

**Statement:**

If $T_{{\ddot{e}}_{ij}}^{-}=\langle \underset{ʝ}{⋀⋀}\underset{ɨ}{⋀⋀}\left(N_{ij}\right),\underset{ʝ}{⋀⋀}\underset{ɨ}{⋀⋀}\left(M_{ij}\right),\underset{ʝ}{⋁}\underset{ɨ}{⋁}\left(L_{ij}\right)\rangle$ and $T_{{\ddot{e}}_{ij}}^{+}$=$\langle \underset{ʝ}{⋁}\underset{ɨ}{⋁}\left(N_{ij}\right),\underset{ʝ}{⋀⋀}\underset{ɨ}{⋀⋀}\left(M_{ij}\right),\underset{ʝ}{⋀⋀}\underset{ɨ}{⋀⋀}\left(L_{ij}\right)\rangle$, then

$T_{{\ddot{e}}_{ij}}^{-}\leq q-\text{ROPF}S_{f}\text{WG}\left(S_{{\ddot{e}}_{11}},S_{{\ddot{e}}_{12}},\ldots .,S_{{\ddot{e}}_{\text{nm}}}\right)\leq T_{{\ddot{e}}_{ij}}^{+}$.

**Proof.** Let $T_{{\ddot{e}}_{ij}}^{-}=\langle \underset{j}{⋀⋀}\underset{i}{⋀⋀}\left(N_{ij}\right),\underset{j}{⋀⋀}\underset{i}{⋀⋀}\left(M_{ij}\right),\underset{j}{⋁}\underset{i}{⋁}\left(L_{ij}\right)\rangle$ and

$T_{{\ddot{e}}_{ij}}^{+}$=$\langle \underset{j}{⋁}\underset{i}{⋁}\left(N_{ij}\right),\underset{j}{⋀⋀}\underset{i}{⋀⋀}\left(M_{ij}\right),\underset{j}{⋀⋀}\underset{i}{⋀⋀}\left(L_{ij}\right)\rangle$. We will prove that

$$T_{{\ddot{e}}_{ij}}^{-}\leq q-\text{ROPF}S_{f}\text{WG}\left(S_{{\ddot{e}}_{11}},S_{{\ddot{e}}_{12}},\ldots .,S_{{\ddot{e}}_{\text{nm}}}\right)\leq T_{{\ddot{e}}_{ij}}^{+}.$$

$$\Rightarrow \underset{j}{⋀⋀}\underset{i}{⋀⋀}\left(N_{ij}\right)\leq N_{ij}\leq \underset{j}{⋁}\underset{i}{⋁}\left(N_{ij}\right)$$

$$\Rightarrow \Pi_{j=1}^{m}{\left(\Pi_{i=1}^{n}{\left(\underset{j}{⋀⋀}\underset{i}{⋀⋀}\left(N_{ij}\right)\right)}^{\omega_{i}}\;\right)}^{\upsilon_{j}}\leq \Pi_{j=1}^{m}{\left(\Pi_{i=1}^{n}{\left(N_{ij}\right)}^{\omega_{i}}\;\right)}^{\upsilon_{j}}\leq \Pi_{j=1}^{m}{\left(\Pi_{i=1}^{n}{\left(\underset{j}{⋁}\underset{i}{⋁}\left(N_{ij}\right)\right)}^{\omega_{i}}\;\right)}^{\upsilon_{j}}$$

$$\Rightarrow {\left({\left(\underset{j}{⋀⋀}\underset{i}{⋀⋀}\left(N_{ij}\right)\right)}^{\sum_{i=1}^{n}\omega_{i}}\;\right)}^{\sum_{j=1}^{m}\upsilon_{j}}\leq \Pi_{j=1}^{m}{\left(\Pi_{i=1}^{n}{\left(N_{ij}\right)}^{\omega_{i}}\;\right)}^{\upsilon_{j}}\leq {\left({\left(\underset{j}{⋁}\underset{i}{⋁}\left(N_{ij}\right)\right)}^{\sum_{i=1}^{n}\omega_{i}}\;\right)}^{\sum_{j=1}^{m}\upsilon_{j}}$$

(11) $$\Rightarrow \underset{j}{⋀⋀}\underset{i}{⋀⋀}\left(N_{ij}\right)\leq \Pi_{j=1}^{m}{\left(\Pi_{i=1}^{n}{\left(N_{ij}\right)}^{\omega_{i}}\;\right)}^{\upsilon_{j}}\leq \underset{j}{⋁}\underset{i}{⋁}\left(N_{ij}\right)$$

Next, for

$$\Rightarrow \underset{j}{⋀⋀}\underset{i}{⋀⋀}\left(M_{ij}\right)\leq M_{ij}\leq \underset{j}{⋁}\underset{i}{⋁}\left(M_{ij}\right)\Rightarrow 1-\underset{j}{⋁}\underset{i}{⋁}\left(M_{ij}^{q}\right)\leq 1-M_{ij}^{q}\leq 1-\underset{j}{⋀⋀}\underset{i}{⋀⋀}\left(M_{ij}^{q}\right)$$

$$\Rightarrow \Pi_{j=1}^{m}{\left(\Pi_{i=1}^{n}{\left(1-\underset{j}{⋁}\underset{i}{⋁}\left(M_{ij}^{q}\right)\right)}^{\omega_{i}}\;\right)}^{\upsilon_{j}}\leq \Pi_{j=1}^{m}{\left(\Pi_{i=1}^{n}{\left(1-M_{ij}^{q}\right)}^{\omega_{i}}\;\right)}^{\upsilon_{j}}\leq \Pi_{j=1}^{m}{\left(\Pi_{i=1}^{n}{\left(1-\underset{j}{⋀⋀}\underset{i}{⋀⋀}\left(M_{ij}^{q}\right)\right)}^{\omega_{i}}\;\right)}^{\upsilon_{j}}$$

$$\Rightarrow {\left({\left(1-\underset{j}{⋁}\underset{i}{⋁}\left(M_{ij}^{q}\right)\right)}^{\sum_{i=1}^{n}\omega_{i}}\;\right)}^{\sum_{j=1}^{m}\upsilon_{j}}\leq \Pi_{j=1}^{m}{\left(\Pi_{i=1}^{n}{\left(1-M_{ij}^{q}\right)}^{\omega_{i}}\;\right)}^{\upsilon_{j}}\leq {\left({\left(1-\underset{j}{⋀⋀}\underset{i}{⋀⋀}\left(M_{ij}^{q}\right)\right)}^{\sum_{i=1}^{n}\omega_{i}}\;\right)}^{\sum_{j=1}^{m}\upsilon_{j}}$$

$$\Rightarrow \left(1-\underset{j}{⋁}\underset{i}{⋁}\left(M_{ij}^{q}\right)\right)\leq \Pi_{j=1}^{m}{\left(\Pi_{i=1}^{n}{\left(1-M_{ij}^{q}\right)}^{\omega_{i}}\;\right)}^{\upsilon_{j}}\leq \left(1-\underset{j}{⋀⋀}\underset{i}{⋀⋀}\left(M_{ij}^{q}\right)\right)$$

$$\Rightarrow 1-\left(1-\underset{j}{⋀⋀}\underset{i}{⋀⋀}\left(M_{ij}^{q}\right)\right)\leq 1-\Pi_{j=1}^{m}{\left(\Pi_{i=1}^{n}{\left(1-M_{ij}^{q}\right)}^{\omega_{i}}\;\right)}^{\upsilon_{j}}\leq 1-\left(1-\underset{j}{⋁}\underset{i}{⋁}\left(M_{ij}^{q}\right)\right)$$

$$\Rightarrow \underset{j}{⋀⋀}\underset{i}{⋀⋀}\left(M_{ij}^{q}\right)\leq 1-\Pi_{j=1}^{m}{\left(\Pi_{i=1}^{n}{\left(1-M_{ij}^{q}\right)}^{\omega_{i}}\;\right)}^{\upsilon_{j}}\leq \underset{j}{⋁}\underset{i}{⋁}\left(M_{ij}^{q}\right)$$

(12) $$\Rightarrow \underset{j}{⋀⋀}\underset{i}{⋀⋀}\left(M_{ij}^{q}\right)\leq \sqrt[{q}]{1-\Pi_{j=1}^{m}{\left(\Pi_{i=1}^{n}{\left(1-M_{ij}^{q}\right)}^{\omega_{i}}\;\right)}^{\upsilon_{j}}}\leq \underset{j}{⋁}\underset{i}{⋁}\left(M_{ij}^{q}\right)$$

And finally,

$$\Rightarrow \underset{j}{⋀⋀}\underset{i}{⋀⋀}\left(L_{ij}\right)\leq L_{ij}\leq \underset{j}{⋁}\underset{i}{⋁}\left(L_{ij}\right)$$

$$\Rightarrow 1-\underset{j}{⋁}\underset{i}{⋁}\left(L_{ij}^{q}\right)\leq 1-L_{ij}^{q}\leq 1-\underset{j}{⋀⋀}\underset{i}{⋀⋀}\left(L_{ij}^{q}\right)$$

$$\Rightarrow \Pi_{j=1}^{m}{\left(\Pi_{i=1}^{n}{\left(1-\underset{j}{⋁}\underset{i}{⋁}\left(L_{ij}^{q}\right)\right)}^{\omega_{i}}\;\right)}^{\upsilon_{j}}\leq \Pi_{j=1}^{m}{\left(\Pi_{i=1}^{n}{\left(1-L_{ij}^{q}\right)}^{\omega_{i}}\;\right)}^{\upsilon_{j}}\leq \Pi_{j=1}^{m}{\left(\Pi_{i=1}^{n}{\left(1-\underset{j}{⋀⋀}\underset{i}{⋀⋀}\left(L_{ij}^{q}\right)\right)}^{\omega_{i}}\;\right)}^{\upsilon_{j}}$$

$$\Rightarrow {\left({\left(1-\underset{j}{⋁}\underset{i}{⋁}\left(L_{ij}^{q}\right)\right)}^{\sum_{i=1}^{n}\omega_{i}}\;\right)}^{\sum_{j=1}^{m}\upsilon_{j}}\leq \Pi_{j=1}^{m}{\left(\Pi_{i=1}^{n}{\left(1-L_{ij}^{q}\right)}^{\omega_{i}}\;\right)}^{\upsilon_{j}}\leq {\left({\left(1-\underset{j}{⋀⋀}\underset{i}{⋀⋀}\left(L_{ij}^{q}\right)\right)}^{\sum_{i=1}^{n}\omega_{i}}\;\right)}^{\sum_{j=1}^{m}\upsilon_{j}}$$

$$\Rightarrow \left(1-\underset{j}{⋁}\underset{i}{⋁}\left(L_{ij}^{q}\right)\right)\leq \Pi_{j=1}^{m}{\left(\Pi_{i=1}^{n}{\left(1-L_{ij}^{q}\right)}^{\omega_{i}}\;\right)}^{\upsilon_{j}}\leq \left(1-\underset{j}{⋀⋀}\underset{i}{⋀⋀}\left(L_{ij}^{q}\right)\right)$$

$$\Rightarrow 1-\left(1-\underset{j}{⋀⋀}\underset{i}{⋀⋀}\left(L_{ij}^{q}\right)\right)\leq 1-\Pi_{j=1}^{m}{\left(\Pi_{i=1}^{n}{\left(1-L_{ij}^{q}\right)}^{\omega_{i}}\;\right)}^{\upsilon_{j}}\leq 1-\left(1-\underset{j}{⋁}\underset{i}{⋁}\left(L_{ij}^{q}\right)\right)$$

$$\Rightarrow \underset{j}{⋀⋀}\underset{i}{⋀⋀}\left(L_{ij}^{q}\right)\leq 1-\Pi_{j=1}^{m}{\left(\Pi_{i=1}^{n}{\left(1-L_{ij}^{q}\right)}^{\omega_{i}}\;\right)}^{\upsilon_{j}}\leq \underset{j}{⋁}\underset{i}{⋁}\left(L_{ij}^{q}\right)$$

(13) $$\Rightarrow \underset{j}{⋀⋀}\underset{i}{⋀⋀}\left(L_{ij}^{q}\right)\leq \sqrt[{q}]{1-\Pi_{j=1}^{m}{\left(\Pi_{i=1}^{n}{\left(1-L_{ij}^{q}\right)}^{\omega_{i}}\;\right)}^{\upsilon_{j}}}\leq \underset{j}{⋁}\underset{i}{⋁}\left(L_{ij}^{q}\right)$$

From eqs (11), (12), and (13), we have

$$\Rightarrow \underset{j}{⋀⋀}\underset{i}{⋀⋀}\left(N_{ij}\right)\leq \Pi_{j=1}^{m}{\left(\Pi_{i=1}^{n}{\left(N_{ij}\right)}^{\omega_{i}}\;\right)}^{\upsilon_{j}}\leq \underset{j}{⋁}\underset{i}{⋁}\left(N_{ij}\right)$$

$$\Rightarrow \underset{j}{⋀⋀}\underset{i}{⋀⋀}\left(M_{ij}^{q}\right)\leq \sqrt[{q}]{1-\Pi_{j=1}^{m}{\left(\Pi_{i=1}^{n}{\left(1-M_{ij}^{q}\right)}^{\omega_{i}}\;\right)}^{\upsilon_{j}}}\leq \underset{j}{⋁}\underset{i}{⋁}\left(M_{ij}^{q}\right)$$

$$\Rightarrow \underset{j}{⋀⋀}\underset{i}{⋀⋀}\left(L_{ij}^{q}\right)\leq \sqrt[{q}]{1-\Pi_{j=1}^{m}{\left(\Pi_{i=1}^{n}{\left(1-L_{ij}^{q}\right)}^{\omega_{i}}\;\right)}^{\upsilon_{j}}}\leq \underset{j}{⋁}\underset{i}{⋁}\left(L_{ij}^{q}\right)$$

Let $\delta =q-\text{ROPF}S_{f}\text{WG}\left(T_{{\ddot{e}}_{11}},T_{{\ddot{e}}_{12}},\ldots .,T_{{\ddot{e}}_{\text{nm}}}\right)=\left(N_{\delta },M_{\delta },\;L_{\delta }\right)$, then by using the score function, we get

$$S\left(\delta \right)=N_{\delta }^{q}-M_{\delta }^{q}-L_{\delta }^{q}+\left(\frac{e^{N_{\delta }^{q}-M_{\delta }^{q}-L_{\delta }^{q}}}{e^{N_{\delta }^{q}-M_{\delta }^{q}-L_{\delta }^{q}}+1}-\frac{1}{2}\right)\pi_{\delta }^{q}$$

$$\leq \left({\left(\underset{j}{⋁}\underset{i}{⋁}\left(N_{ij}\right)\right)}^{q}-{\left(\underset{j}{⋀⋀}\underset{i}{⋀⋀}\left(M_{ij}\right)\right)}^{q}-{\left(\underset{j}{⋀⋀}\underset{i}{⋀⋀}\left(L_{ij}\right)\right)}^{q}\right)\;+\left(\frac{e^{\left({\left(\underset{j}{⋀⋀}\underset{i}{⋀⋀}\left(N_{ij}\right)\right)}^{q}-{\left(\underset{j}{⋀⋀}\underset{i}{⋀⋀}\left(M_{ij}\right)\right)}^{q}-{\left(\underset{j}{⋁}\underset{i}{⋁}\left(L_{ij}\right)\right)}^{q}\right)}}{e^{\left({\left(\underset{j}{⋀⋀}\underset{i}{⋀⋀}\left(N_{ij}\right)\right)}^{q}-{\left(\underset{j}{⋀⋀}\underset{i}{⋀⋀}\left(M_{ij}\right)\right)}^{q}-{\left(\underset{j}{⋁}\underset{i}{⋁}\left(L_{ij}\right)\right)}^{q}\right)}+1}-\frac{1}{2}\right)\pi_{T_{{\ddot{e}}_{ij}}^{+}}^{q}$$

$=\;S\left(T_{{\ddot{e}}_{ij}}^{+}\right)\;=\;S\left(\delta \right)\leq S\left(T_{{\ddot{e}}_{ij}}^{+}\right)\;\text{and}$

$$S\left(\delta \right)=N_{\delta }^{q}-M_{\delta }^{q}-L_{\delta }^{q}+\left(\frac{e^{N_{\delta }^{q}-M_{\delta }^{q}-L_{\delta }^{q}}}{e^{N_{\delta }^{q}-M_{\delta }^{q}-L_{\delta }^{q}}+1}-\frac{1}{2}\right)\pi_{\delta }^{q}\geq \left({\left(\underset{j}{⋀⋀}\underset{i}{⋀⋀}\left(N_{ij}\right)\right)}^{q}-{\left(\underset{j}{⋀⋀}\underset{i}{⋀⋀}\left(M_{ij}\right)\right)}^{q}-{\left(\underset{j}{⋁}\underset{i}{⋁}\left(L_{ij}\right)\right)}^{q}\right)\;$$

$\left(\frac{e^{\left({\left(\underset{j}{⋀⋀}\underset{i}{⋀⋀}\left(N_{ij}\right)\right)}^{q}-{\left(\underset{j}{⋀⋀}\underset{i}{⋀⋀}\left(M_{ij}\right)\right)}^{q}-{\left(\underset{j}{⋁}\underset{i}{⋁}\left(L_{ij}\right)\right)}^{q}\right)}}{e^{\left({\left(\underset{j}{⋀⋀}\underset{i}{⋀⋀}\left(N_{ij}\right)\right)}^{q}-{\left(\underset{j}{⋀⋀}\underset{i}{⋀⋀}\left(M_{ij}\right)\right)}^{q}-{\left(\underset{j}{⋁}\underset{i}{⋁}\left(L_{ij}\right)\right)}^{q}\right)}+1}-\frac{1}{2}\right)\pi_{T_{{\ddot{e}}_{ij}}^{-}}^{q}=\;S\left(T_{{\ddot{e}}_{ij}}^{-}\right)\;$= $S\left(\delta \right)\geq S\left(T_{{\ddot{e}}_{ij}}^{-}\right)$.

From the above inequality, the following three cases arise.

Case 1. By the comparison of two $q-\text{ROPF}S_{t}N_{s}$, if $S\left(\delta \right)<S\left(T_{{\ddot{e}}_{ij}}^{+}\right)$ and $S\left(\delta \right)>S\left(T_{{\ddot{e}}_{ij}}^{-}\right)$, then we get

$T_{{\ddot{e}}_{ij}}^{-}\leq q-\text{ROPF}S_{f}\text{WG}\left(S_{{\ddot{e}}_{11}},S_{{\ddot{e}}_{12}},\ldots .,S_{{\ddot{e}}_{\text{nm}}}\right)\leq T_{{\ddot{e}}_{ij}}^{+}$.

Case 2. If $S\left(\delta \right)=S\left(T_{{\ddot{e}}_{ɨʝ}}^{+}\right)$, then

$$S\left(\delta \right)=N_{\delta }^{q}-M_{\delta }^{q}-L_{\delta }^{q}+\left(\frac{e^{N_{\delta }^{q}-M_{\delta }^{q}-L_{\delta }^{q}}}{e^{N_{\delta }^{q}-M_{\delta }^{q}-L_{\delta }^{q}}+1}-\frac{1}{2}\right)\pi_{\delta }^{q}$$

$$={\left(\underset{j}{⋁}\underset{i}{⋁}\left(N_{ij}\right)\right)}^{q}-{\left(\underset{j}{⋀⋀}\underset{i}{⋀⋀}\left(M_{ɨʝ}\right)\right)}^{q}-{\left(\underset{j}{⋀⋀}\underset{i}{⋀⋀}\left(L_{ij}\right)\right)}^{q}+\left(\frac{e^{\left({\left(\underset{j}{⋀⋀}\underset{i}{⋀⋀}\left(N_{ij}\right)\right)}^{q}-{\left(\underset{j}{⋀⋀}\underset{i}{⋀⋀}\left(M_{ij}\right)\right)}^{q}-{\left(\underset{j}{⋁}\underset{i}{⋁}\left(L_{ij}\right)\right)}^{q}\right)}}{e^{\left({\left(\underset{j}{⋀⋀}\underset{i}{⋀⋀}\left(N_{ij}\right)\right)}^{q}-{\left(\underset{j}{⋀⋀}\underset{i}{⋀⋀}\left(M_{ij}\right)\right)}^{q}-{\left(\underset{j}{⋁}\underset{i}{⋁}\left(L_{ij}\right)\right)}^{q}\right)}+1}-\frac{1}{2}\right)\pi_{T_{{\ddot{e}}_{ij}}^{+}}^{q}$$

We get from the above inequality

$N_{\delta }=\underset{j}{⋁}\underset{i}{⋁}\left(N_{ij}\right)$, $M_{\delta }=\underset{j}{⋀⋀}\underset{i}{⋀⋀}\left(M_{ij}\right)$, $L_{\delta }=\underset{j}{⋀⋀}\underset{i}{⋀⋀}\left(L_{ij}\right)$. Thus $\pi_{\delta }^{q}=\pi_{A_{{\ddot{e}}_{ij}}^{+}}^{q}$. We get

$q-\text{ROPF}S_{f}\text{WG}\left(S_{{\ddot{e}}_{11}},S_{{\ddot{e}}_{12}},\ldots .,S_{{\ddot{e}}_{\text{nm}}}\right)=v_{{\ddot{e}}_{ij}}^{+}$.

Case 3. If $S\left(\delta \right)=S\left(T_{{\ddot{e}}_{ɨʝ}}^{-}\right)$, then

$S\left(\delta \right)=N_{\delta }^{q}-M_{\delta }^{q}-L_{\delta }^{q}+\left(\frac{e^{N_{\delta }^{q}-M_{\delta }^{q}-L_{\delta }^{q}}}{e^{N_{\delta }^{q}-M_{\delta }^{q}-L_{\delta }^{q}}+1}-\frac{1}{2}\right)\pi_{\delta }^{q}$
  $$={\left(\underset{j}{⋀⋀}\underset{i}{⋀⋀}\left(N_{ij}\right)\right)}^{q}-{\left(\underset{j}{⋀⋀}\underset{i}{⋀⋀}\left(M_{ij}\right)\right)}^{q}-{\left(\underset{j}{⋁}\underset{i}{⋁}\left(L_{ij}\right)\right)}^{q}+\left(\frac{e^{\left({\left(\underset{j}{⋀⋀}\underset{i}{⋀⋀}\left(N_{ij}\right)\right)}^{q}-{\left(\underset{j}{⋀⋀}\underset{i}{⋀⋀}\left(M_{ij}\right)\right)}^{q}-{\left(\underset{j}{⋁}\underset{i}{⋁}\left(L_{ij}\right)\right)}^{q}\right)}}{e^{\left({\left(\underset{j}{⋀⋀}\underset{i}{⋀⋀}\left(N_{ij}\right)\right)}^{q}-{\left(\underset{j}{⋀⋀}\underset{i}{⋀⋀}\left(M_{ij}\right)\right)}^{q}-{\left(\underset{j}{⋁}\underset{i}{⋁}\left(L_{ij}\right)\right)}^{q}\right)}+1}-\frac{1}{2}\right)\pi_{T_{{\ddot{e}}_{ij}}^{-}}^{q}$$

We get from the above inequality.

$N_{\delta }=\underset{j}{⋀⋀}\underset{i}{⋀⋀}\left(N_{ij}\right),$ $M_{\delta }=\underset{j}{⋀⋀}\underset{i}{⋀⋀}\left(M_{ij}\right)$, $L_{\delta }=\underset{j}{⋁}\underset{i}{⋁}\left(L_{ij}\right)$. Thus $\pi_{\delta }^{q}=\pi_{A_{{\ddot{e}}_{ij}}^{-}}^{q}$. We get

$$q-\text{ROPF}S_{f}\text{WG}\left(S_{{\ddot{e}}_{11}},S_{{\ddot{e}}_{12}},\ldots .,S_{{\ddot{e}}_{\text{nm}}}\right)=T_{{\ddot{e}}_{ij}}^{-}.$$

Hence, $T_{{\ddot{e}}_{ij}}^{-}\leq q-\text{ROPF}S_{f}\text{WG}\left(S_{{\ddot{e}}_{11}},S_{{\ddot{e}}_{12}},\ldots .,S_{{\ddot{e}}_{\text{nm}}}\right)\leq T_{{\ddot{e}}_{ij}}^{+}.$

Property 3 **Monotonicity**

**Statement:** Assume the collection of two $q-\text{ROPF}S_{t}N_{s}$ $T_{{\ddot{e}}_{ij}}=\left(N_{ij},M_{ij},\;L_{ij}\right)$ and

$N_{{\ddot{e}}_{ij}}=\left({₩₩}_{\text{ij}},{₲}_{\text{ij}},{\;₱}_{\text{ij}}\right),\;\text{if}\;N_{ij}\leq {₩₩}_{\text{ij}},\;M_{ij}\leq {₲}_{\text{ij}},\;L_{ij}\geq {₱}_{\text{ij}}$, then

q−ROPF$S_{f}$WA $(T_{{\ddot{e}}_{11}},T_{{\ddot{e}}_{12}},\ldots .,T_{{\ddot{e}}_{\text{nm}}})\;$≤q− ROPF$S_{f}$WA $({₦}_{{\ddot{e}}_{11}},{₦}_{{\ddot{e}}_{12}},\ldots .,{₦}_{{\ddot{e}}_{\text{nm}}}).$

q−ROPF$S_{f}$OWA $(T_{{\ddot{e}}_{11}},T_{{\ddot{e}}_{12}},\ldots .,T_{{\ddot{e}}_{\text{nm}}})\;$≤ $q-\text{ROPF}S_{f}\text{OWA}\;\left({₦}_{{\ddot{e}}_{11}},{₦}_{{\ddot{e}}_{12}},\ldots .,{₦}_{{\ddot{e}}_{\text{nm}}}\right).$

**Proof.** Consider two $q-\text{ROPF}S_{t}N_{s}$ $T_{{\ddot{e}}_{ij}}=\left(N_{ij},M_{ij},\;L_{v}\right)$ and $N_{{\ddot{e}}_{ij}}=\left(₩{₩}_{ij},{₲}_{ij},\;{₱}_{ij}\right)$, if

$N_{ij}\leq {₩₩}_{\text{ij}},\;M_{ij}\leq {₲}_{\text{ij}},\;L_{ij}\geq {₱}_{\text{ij}}$, then

$$\Rightarrow N_{ij}\leq ₩{₩}_{ij}$$

$$\Rightarrow \Pi_{i=1}^{n}{\left(N_{ij}\right)}^{\omega_{i}}\leq \Pi_{i=1}^{n}{\left(₩{₩}_{ij}\right)}^{\omega_{i}}$$

(14) $$\Rightarrow \Pi_{j=1}^{m}{\left(\Pi_{i=1}^{n}{\left(N_{ij}\right)}^{\omega_{i}}\right)}^{\upsilon_{j}}\leq \Pi_{j=1}^{m}{\left(\Pi_{i=1}^{n}{\left(₩{₩}_{ij}\right)}^{\omega_{i}}\right)}^{\upsilon_{j}}$$

Next for

$$\Rightarrow M_{ij}\leq {₲}_{ij}$$

$$\Rightarrow 1-{₲}_{ij}\leq 1-M_{ij}$$

$$\Rightarrow 1-{₲}_{ij}^{q}\leq 1-M_{ij}^{q}$$

$$\Rightarrow \Pi_{j=1}^{m}{\left(\Pi_{i=1}^{n}{\left(1-{₲}_{ij}^{q}\right)}^{\omega_{i}}\right)}^{\upsilon_{j}}\leq \Pi_{j=1}^{m}{\left(\Pi_{i=1}^{n}{\left(1-M_{ij}^{q}\right)}^{\omega_{i}}\right)}^{\upsilon_{j}}$$

$$\Rightarrow 1-\Pi_{j=1}^{m}{\left(\Pi_{i=1}^{n}{\left(1-M_{ij}^{q}\right)}^{\omega_{i}}\right)}^{\upsilon_{j}}\leq 1-\Pi_{j=1}^{m}{\left(\Pi_{i=1}^{n}{\left(1-{₲}_{ij}^{q}\right)}^{\omega_{i}}\right)}^{\upsilon_{j}}$$

(15) $$\Rightarrow \sqrt[{q}]{\;1-\Pi_{j=1}^{m}{\left(\Pi_{i=1}^{n}{\left(1-M_{ij}^{q}\right)}^{\omega_{i}}\right)}^{\upsilon_{j}}}\leq \sqrt[{q}]{1-\Pi_{j=1}^{m}{\left(\Pi_{i=1}^{n}{\left(1-{₲}_{ij}^{q}\right)}^{\omega_{i}}\right)}^{\upsilon_{j}}}$$

And finally, for,

$$\Rightarrow L_{ij}\geq {₱}_{ij}$$

$$\Rightarrow 1-{₱}_{ij}\geq 1-L_{ij}$$

$$\Rightarrow 1-{₱}_{ij}^{q}\geq 1-L_{ij}^{q}$$

$$\Rightarrow \Pi_{j=1}^{m}{\left(\Pi_{i=1}^{n}{\left(1-{₱}_{ij}^{q}\right)}^{\omega_{i}}\right)}^{\upsilon_{j}}\geq \Pi_{j=1}^{m}{\left(\Pi_{i=1}^{n}{\left(1-L_{ij}^{q}\right)}^{\omega_{i}}\right)}^{\upsilon_{j}}$$

$$\Rightarrow 1-\Pi_{j=1}^{m}{\left(\Pi_{i=1}^{n}{\left(1-L_{ij}^{q}\right)}^{\omega_{i}}\right)}^{\upsilon_{j}}\geq 1-\Pi_{j=1}^{m}{\left(\Pi_{i=1}^{n}{\left(1-{₱}_{ij}^{q}\right)}^{\omega_{i}}\right)}^{\upsilon_{j}}$$

(16) $$\Rightarrow \sqrt[{q}]{\;1-\Pi_{j=1}^{m}{\left(\Pi_{i=1}^{n}{\left(1-L_{ij}^{q}\right)}^{\omega_{i}}\right)}^{\upsilon_{j}}}\geq \sqrt[{q}]{1-\Pi_{j=1}^{m}{\left(\Pi_{i=1}^{n}{\left(1-{₱}_{ij}^{q}\right)}^{\omega_{i}}\right)}^{\upsilon_{j}}}$$

Let $\delta_{T}=q-\text{ROPF}S_{f}\text{WG}\left(T_{{\ddot{e}}_{11}},T_{{\ddot{e}}_{12}},\ldots .,T_{{\ddot{e}}_{\text{nm}}}\right)=\left(N_{\delta_{T}},M_{\delta_{T}},\;L_{\delta_{T}}\right)$ and

$$\delta_{N}=q-\text{ROPF}S_{f}\text{WG}\left(N_{{\ddot{e}}_{11}},N_{{\ddot{e}}_{12}},\ldots .,N_{{\ddot{e}}_{\text{nm}}}\right)=\left(₩{₩}_{\delta_{₦}},{₲}_{\delta_{₦}},\;{₱}_{\delta_{₦}}\right)$$

Now, from eqs (14), (15), and (16), we have

$$N_{\delta_{T}}\leq ₩{₩}_{\delta_{N}},M_{\delta_{T}}\leq {₲}_{\delta_{N}}\;\text{and}\;L_{\delta_{T}}\geq {₱}_{\delta_{N}}$$

Then from the score function, $S\left(\delta_{T}\right)<S\left(\delta_{N}\right)$,

The above condition results in the occurrences of the following scenarios:

Case 1. When comparing two $q-\text{ROPF}S_{t}N_{s}$, if $S\left(\delta_{T}\right)\;\text{is}\;\text{less}\;\text{than}\;S\left(\delta_{N}\right)$, then

$q-\text{ROPF}S_{f}\text{WG}\left(T_{{\ddot{e}}_{11}},T_{{\ddot{e}}_{12}},\ldots .,T_{{\ddot{e}}_{\text{nm}}}\right)<\;q-\text{ROPF}S_{f}\text{WG}\left(N_{{\ddot{e}}_{11}},N_{{\ddot{e}}_{12}},\ldots .,N_{{\ddot{e}}_{\text{nm}}}\right)$.

Case 2. If two $q-\text{ROPF}S_{t}N_{s}$ are equal, $S\left(\delta_{T}\right)=S\left(\delta_{N}\right)$

$$S\left(\delta_{T}\right)=N_{\delta_{T}}^{q}-M_{\delta_{T}}^{q}-L_{\delta_{T}}^{q}+\left(\frac{e^{N_{\delta_{T}}^{q}-M_{\delta_{T}}^{q}-L_{\delta_{T}}^{q}}}{e^{N_{\delta_{T}}^{q}-M_{\delta_{T}}^{q}-L_{\delta_{T}}^{q}}+1}-\frac{1}{2}\right)\pi_{\delta_{T}}^{q}$$

$$=N_{\delta_{N}}^{q}-M_{\delta_{N}}^{q}-L_{\delta_{N}}^{q}+\left(\frac{e^{N_{\delta_{N}}^{q}-M_{\delta_{N}}^{q}-L_{\delta_{N}}^{q}}}{e^{N_{\delta_{N}}^{q}-M_{\delta_{N}}^{q}-L_{\delta_{N}}^{q}}+1\;}-\frac{1}{2}\right)\pi_{\delta_{N}}^{q}=\;S\left(\delta_{N}\right),$$

$$\text{Since},\;N_{\delta_{T}}={₩₩}_{\delta_{N}},M_{\delta_{T}}={₲}_{\delta_{N}}\;\text{and}\;L_{\delta_{T}}={₱}_{\delta_{N}}$$

$$\Rightarrow \pi_{\delta_{T}}^{q}=\pi_{\delta_{N}}^{q}$$

$$\Rightarrow \left(N_{\delta_{T}},M_{\delta_{T}},\;L_{\delta_{T}}\right)=\left(₩{₩}_{\delta_{N}},{₲}_{\delta_{N}},{₱}_{\delta_{N}}\right)$$

Hence, $q-\text{ROPF}S_{f}\text{WG}\left(T_{{\ddot{e}}_{11}},T_{{\ddot{e}}_{12}},\ldots .,T_{{\ddot{e}}_{\text{nm}}}\right)\leq \;q-\text{ROPF}S_{f}\text{WG}\left(N_{{\ddot{e}}_{11}},N_{{\ddot{e}}_{12}},\ldots .,N_{{\ddot{e}}_{\text{nm}}}\right)$.

**Justification of Monotonicity via Derivative Logic:**

Consider the aggregation components in the $q-\text{ROPF}S_{f}\text{WG}$ operator, such as the product terms:

$$f\;\left(T_{1},T_{2},\ldots .,T_{n}\right)=\prod_{i=1}^{n}T_{i}^{\omega_{i}}$$

This function is monotonically increasing in each $T_{i}$∈ [0,1] because

$$\frac{\partial f}{\partial A_{i}}=\;\omega_{i}T_{i}^{\omega_{i}-1}\cdot \prod_{i\neq j}T_{j}^{\omega_{j}}\geq 0,\;T_{i}\geq 0$$

Since each exponent $\omega_{i}$ ≥0 and all $T_{i}$∈ [0,1], the derivative is non-negative, confirming that increasing any $A_{i}$ (with others fixed) does not decrease the value of the product. Thus, this part of the aggregation preserves monotonicity**.**

Similarly, in the expression:

$$g\;\left(H_{1},H_{2},\ldots .,H_{n}\right)=1-\left(\prod_{i=1}^{n}{\left(1-H_{i}^{q}\right)}^{\omega_{i}}\right)$$

The term inside the product is decreasing in each $H_{i}$, but since we take 1 minus the product, the whole function becomes increasing in each $H_{i}$. This can be justified via:

$$\frac{\partial g}{\partial B_{i}}\;=\;q\cdot \;\omega_{i}\cdot H_{i}^{q-1}\cdot \prod_{i\neq j}T_{j}^{\omega_{j}}\cdot \;\left(\text{positive}\;\text{factor}\right)\;\geq 0$$

Hence, the whole $q-\text{ROPF}S_{f}\;\text{WG}$ operator is monotonic increasing in $T_{i}$, $H_{i}$ and monotonic decreasing in $L_{i}$ due to similar derivative behaviour.

From the detailed algebraic inequalities already presented in eqs (15), (16), and (17), we conclude that the components of the aggregation functions exhibit monotonic behaviour either increasing or decreasing as appropriate based on their mathematical formulation. This is further confirmed by analysing their partial derivatives, which are non-negative or non-positive depending on the respective input variables. Such derivative behaviour ensures that the functions respond predictably to changes in inputs. Consequently, the $q-\text{ROPF}S_{f}\text{WG}$ and $q-\text{ROPF}S_{f}\text{OWG}$ operators preserve monotonicity with respect to increasing or decreasing values of the input elements, validating the consistency and reliability of these aggregation mechanisms in decision-making processes.

Property 4 **Shift Invariance:**

**Statement:** Consider the collection of two q-RON$\text{PF}N_{s}$ $T_{{\ddot{e}}_{ij}}=\left(N_{ij},M_{ij},\;L_{ij}\right)$ and $m_{1}$, then

$q-\text{ROPF}S_{f}\text{WG}\left(T_{{\ddot{e}}_{11}}⊗N_{\ddot{e}},T_{{\ddot{e}}_{12}}⊗N_{\ddot{e}},\ldots .,T_{{\ddot{e}}_{\text{nm}}}⊗N_{\ddot{e}}\right)=q-\text{ROPF}S_{f}\text{WG}\left(T_{{\ddot{e}}_{11}},T_{{\ddot{e}}_{12}},\ldots .,T_{{\ddot{e}}_{\text{nm}}}\right)⊗N_{\ddot{e}}$.

$q-\;\text{ROPF}S_{f}\text{OWG}\left(T_{{\ddot{e}}_{11}}⊗N_{\ddot{e}},T_{{\ddot{e}}_{12}}⊗N,\ldots .,T_{{\ddot{e}}_{\text{nm}}}⊗N_{\ddot{e}}\right)=q-\text{ROPF}S_{f}\text{OWG}\left(T_{{\ddot{e}}_{11}},T_{{\ddot{e}}_{12}},\ldots .,T_{{\ddot{e}}_{\text{nm}}}\right)⊗N$_._

**Proof.** Consider two q-ROPF$S_{f}N_{s}\;T_{{\ddot{e}}_{ij}}=\left(N_{ij},M_{ij},\;L_{ij}\right)$ and $N_{\ddot{e}}=\left(₩₩,\;₲,\;₱\right)$, we know that

$$q-\text{ROPF}S_{f}\text{WG}\left(T_{{\ddot{e}}_{11}}⊗N_{\ddot{e}},T_{{\ddot{e}}_{12}}⊗N_{\ddot{e}},\ldots .,T_{{\ddot{e}}_{\text{nm}}}⊗N_{\ddot{e}}\right)={⊗}_{j=1}^{m}{\left({⊗}_{i=1}^{n}{\left(T_{{\ddot{e}}_{nm}}⊗N_{\ddot{e}}\right)}^{\omega_{i}}\right)}^{\upsilon_{j}}$$

We know that $N_{\ddot{e}}=\left(₩₩,\;₲,\;₱\right)$

$=\left(\begin{array}{c} \;\Pi_{j=1}^{m}{\left(\Pi_{i=1}^{n}N_{ij}^{\omega_{i}}\;\right)}^{\upsilon_{j}},\\ \sqrt[{q}]{1-\prod_{j=1}^{m}{\left(\prod_{i=1}^{n}{\left(1-M_{ij}^{q}\right)}^{\omega_{i}}\right)}^{\upsilon_{j}}},\\ \sqrt[{q}]{1-\prod_{j=1}^{m}{\left(\prod_{i=1}^{n}{\left(1-L_{ij}^{q}\right)}^{\omega_{i}}\right)}^{\upsilon_{j}}} \end{array}\right)⊗\left(₩₩,\;₲,\;₱\right)=\;q-\text{ROPF}S_{f}\text{WG}\left(T_{{\ddot{e}}_{11}},T_{{\ddot{e}}_{12}},\ldots .,T_{{\ddot{e}}_{\text{nm}}}\right)⊗N_{\ddot{e}}$.

Similarly, we can prove for $q-\text{ROPF}S_{f}\text{OWG}$ operator. For this we consider two $T_{{\ddot{e}}_{\sigma ij}}=\left(N_{\sigma ij},M_{\sigma ij},\;L_{\sigma ij}\right)$ and $N_{\ddot{e}}=\left(₩₩,\;₲,\;₱\right)$ are q-ROPF$S_{f}N_{s}$.

$$T_{{\ddot{e}}_{\sigma ij}}⊗N_{\ddot{e}}=\left(N_{11}₩₩,\sqrt[{q}]{\left(1-M_{\sigma ij}^{q}\right)\left(1-{₲}^{q}\right)},\sqrt[{q}]{\left(1-L_{\sigma ij}^{q}\right)\left(1-{₱}^{q}\right)}\right)$$

$$q-\text{ROPF}S_{f}\text{OWG}\left(T_{{\ddot{e}}_{11}}⊗N_{\ddot{e}},T_{{\ddot{e}}_{12}}⊗N_{\ddot{e}},\ldots .,T_{{\ddot{e}}_{\text{nm}}}⊗N_{\ddot{e}}\right)={⊗}_{j=1}^{m}{\left({⊗}_{i=1}^{n}{\left(T_{{\ddot{e}}_{\sigma nm}}⊗N_{\ddot{e}}\right)}^{\omega_{i}}\right)}^{\upsilon_{j}}$$

$$=\left(\begin{array}{c} \Pi_{j=1}^{m}{\left(\Pi_{i=1}^{n}N_{\sigma ij}^{\omega_{i}}\;₩{₩}^{\omega_{i}}\right)}^{\upsilon_{j}},\sqrt[{q}]{1-\prod_{j=1}^{m}{\left(\prod_{i=1}^{n}{\left(1-M_{\sigma ij}^{q}\right)}^{\omega_{i}}{\left(1-{₲}^{q}\right)}^{\omega_{i}}\right)}^{\upsilon_{j}}},\\ \sqrt[{q}]{1-\prod_{j=1}^{m}{\left(\prod_{i=1}^{n}{\left(1-L_{\sigma ij}^{q}\right)}^{\omega_{i}}{\left(1-{₱}^{q}\right)}^{\omega_{i}}\right)}^{\upsilon_{j}}} \end{array}\right)$$

$=\left(\begin{array}{c} \;\Pi_{j=1}^{m}{\left(\Pi_{i=1}^{n}N_{\sigma ij}^{\omega_{i}}\;\right)}^{\upsilon_{j}},\\ \sqrt[{q}]{1-\prod_{j=1}^{m}{\left(\prod_{i=1}^{n}{\left(1-M_{\sigma ij}^{q}\right)}^{\omega_{i}}\right)}^{\upsilon_{j}}},\\ \sqrt[{q}]{1-\prod_{j=1}^{m}{\left(\prod_{i=1}^{n}{\left(1-L_{\sigma ij}^{q}\right)}^{\omega_{i}}\right)}^{\upsilon_{j}}} \end{array}\right)⊗\left(₩₩,\;₲,\;₱\right)$.

$=\;q-\text{ROPF}S_{f}\text{OWG}\left(T_{{\ddot{e}}_{11}},T_{{\ddot{e}}_{12}},\ldots .,T_{{\ddot{e}}_{\text{nm}}}\right)⊗N_{\ddot{e}}$

Hence the result is hold.

Property 5 **Homogeneity**

**Statement:** Consider a real number λ ≥ 0, then

$q-\text{ROPF}S_{f}\text{WG}\left(\lambda T_{{\ddot{e}}_{11}},\lambda T_{{\ddot{e}}_{12}},\ldots .,\lambda T_{{\ddot{e}}_{\text{nm}}}\right)=\lambda q-\text{ROPF}S_{f}\text{WG}\left(T_{{\ddot{e}}_{11}},T_{{\ddot{e}}_{12}},\ldots .,T_{{\ddot{e}}_{\text{nm}}}\right)$.

$q-\text{ROPF}S_{f}\text{OWG}\left(\lambda T_{{\ddot{e}}_{11}},\lambda T_{{\ddot{e}}_{12}},\ldots .,\lambda T_{{\ddot{e}}_{\text{nm}}}\right)=\lambda q-\text{ROPF}S_{f}\text{OWG}\left(T_{{\ddot{e}}_{11}},T_{{\ddot{e}}_{12}},\ldots .,T_{{\ddot{e}}_{\text{nm}}}\right)$.

**Proof.** Consider q-ROPF$S_{f}N_{s}\;T_{{\ddot{e}}_{ɨʝ}}=\left(N_{ɨʝ},M_{ɨʝ},\;L_{ɨʝ}\right)$ For real number λ≥0, we have

$$T_{ij}^{\lambda }=\left({\left(N_{ij}\right)}^{\lambda },\sqrt[{q}]{1-{\left(1-{\left(M_{ij}\right)}^{q}\right)}^{\lambda }},\sqrt[{q}]{1-{\left(1-{\left(L_{ij}\right)}^{q}\right)}^{\lambda }}\right)$$

$$q-\text{ROPF}S_{f}\text{WG}\left(\lambda T_{{\ddot{e}}_{11}},\lambda T_{{\ddot{e}}_{12}},\ldots .,\lambda T_{{\ddot{e}}_{\text{nm}}}\right)\;=\left(\begin{array}{c} \Pi_{j=1}^{m}{\left(\Pi_{i=1}^{n}N_{ij}^{\lambda \omega_{i}}\right)}^{\upsilon_{j}},\\ \sqrt[{q}]{1-\prod_{j=1}^{m}{\left(\prod_{i=1}^{n}{\left(1-M_{ij}^{q}\right)}^{\lambda \omega_{i}}\right)}^{\upsilon_{j}}},\\ \sqrt[{q}]{1-\prod_{j=1}^{m}{\left(\prod_{i=1}^{n}{\left(1-L_{ij}^{q}\right)}^{\lambda \omega_{i}}\right)}^{\upsilon_{j}}} \end{array}\right)$$

$$=\left(\begin{array}{c} {\left(\Pi_{j=1}^{m}{\left(\Pi_{i=1}^{n}N_{ij}^{\omega_{i}}\right)}^{\upsilon_{j}}\right)}^{\lambda },\\ \sqrt[{q}]{1-{\left(\prod_{j=1}^{m}{\left(\prod_{i=1}^{n}{\left(1-M_{ij}^{q}\right)}^{\omega_{i}}\right)}^{\upsilon_{j}}\right)}^{\lambda }},\\ \sqrt[{q}]{1-{\left(\prod_{j=1}^{m}{\left(\prod_{i=1}^{n}{\left(1-L_{ij}^{q}\right)}^{\omega_{i}}\right)}^{\upsilon_{j}}\right)}^{\lambda }} \end{array}\right)=\;\lambda q-\text{ROPF}S_{f}\text{WG}\left(T_{{\ddot{e}}_{11}},T_{{\ddot{e}}_{12}},\ldots .,T_{{\ddot{e}}_{\text{nm}}}\right).$$

Now for $q-\text{ROPF}S_{f}\text{OWG}$ operator, we consider λ≥0 and $T_{{\ddot{e}}_{ɨʝ}}=\left(N_{ɨʝ},M_{ɨʝ},\;L_{ɨʝ}\right)$ q-ROPF$S_{f}N_{s}$, then

$$T_{\sigma ij}^{\lambda }=\left({\left(N_{\sigma ij}\right)}^{\lambda },\sqrt[{q}]{1-{\left(1-{\left(M_{\sigma ij}\right)}^{q}\right)}^{\lambda }},\sqrt[{q}]{1-{\left(1-{\left(L_{\sigma ij}\right)}^{q}\right)}^{\lambda }}\right)$$

$$q-\text{ROPF}S_{f}\text{OWG}\left(\lambda T_{{\ddot{e}}_{11}},\lambda T_{{\ddot{e}}_{12}},\ldots .,\lambda T_{{\ddot{e}}_{\text{nm}}}\right)\;=\left(\begin{array}{c} \Pi_{j=1}^{m}{\left(\Pi_{i=1}^{n}N_{\sigma ij}^{\lambda \omega_{i}}\right)}^{\upsilon_{j}},\\ \sqrt[{q}]{1-\prod_{j=1}^{m}{\left(\prod_{i=1}^{n}{\left(1-M_{\sigma ij}^{q}\right)}^{\lambda \omega_{i}}\right)}^{\upsilon_{j}}},\\ \sqrt[{q}]{1-\prod_{j=1}^{m}{\left(\prod_{i=1}^{n}{\left(1-L_{\sigma ij}^{q}\right)}^{\lambda \omega_{i}}\right)}^{\upsilon_{j}}} \end{array}\right)$$

$$=\left(\begin{array}{c} {\left(\Pi_{j=1}^{m}{\left(\Pi_{i=1}^{n}N_{\sigma ij}^{\omega_{i}}\right)}^{\upsilon_{j}}\right)}^{\lambda },\\ \sqrt[{q}]{1-{\left(\prod_{j=1}^{m}{\left(\prod_{i=1}^{n}{\left(1-M_{\sigma ij}^{q}\right)}^{\omega_{i}}\right)}^{\upsilon_{j}}\right)}^{\lambda }},\\ \sqrt[{q}]{1-{\left(\prod_{j=1}^{m}{\left(\prod_{i=1}^{n}{\left(1-L_{\sigma ij}^{q}\right)}^{\omega_{i}}\right)}^{\upsilon_{j}}\right)}^{\lambda }} \end{array}\right)$$

$\lambda q-\text{ROPF}S_{f}\text{OWG}\left(T_{{\ddot{e}}_{11}},T_{{\ddot{e}}_{12}},\ldots .,T_{{\ddot{e}}_{\text{nm}}}\right)$.

Hence, the result is hold.

### Some special cases

In this section, we discuss some special cases to examine the effect of different restrictions.

i. The proposed AOs degenerate into $\text{IF}S_{f}$ aggregation operators, when the power of membership, non-membership = 1 (*q* =1) and neutral degree=0 (*q* =0).
ii. In this case, AOs are reduced to $P_{y}FS_{f}$ aggregation operators, when the power of membership, non-membership = 2 (*q* =2) and neutral degree=0 (*q* =0).
iii. The proposed AOs degenerate into $q-\text{ROF}S_{f}$ aggregation operators, when the power of membership, non-membership ≥3 (*q* ≥3) and neutral degree=0 (*q* =0).
iv. The proposed AOs degenerate into $\text{FF}S_{f}$ aggregation operators, when the power of membership, non-membership = 3 (*q* =3) and neutral degree=0 (*q* =0).

**Fig. 5:**
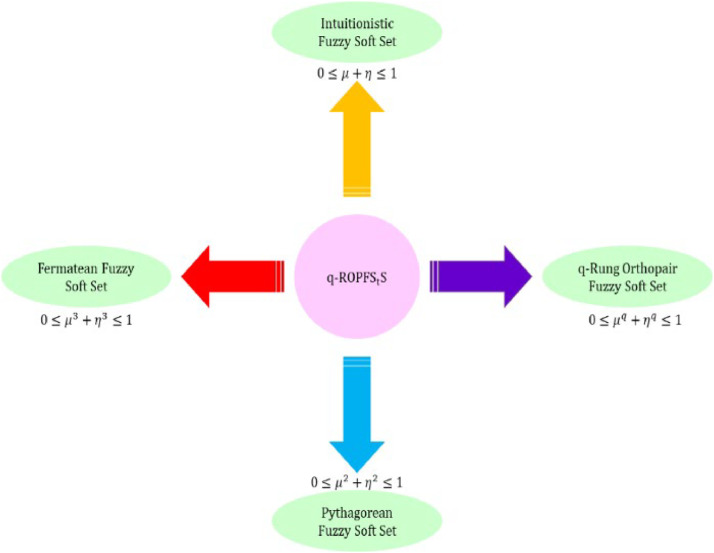
Special cases and proposed operator.

The special cases and proposed operators are expressed in Fig. 5.

From the above, we obtain that $\text{IF}S_{f}$WG, $P_{y}FS_{f}$WG, $q-\text{ROF}S_{f}$WG and $\text{FF}S_{f}$WG are the special cases of the proposed $q-\text{ROPF}S_{f}$WG operator.

### MADM under q-ROPF soft information

**Fig. 6:**
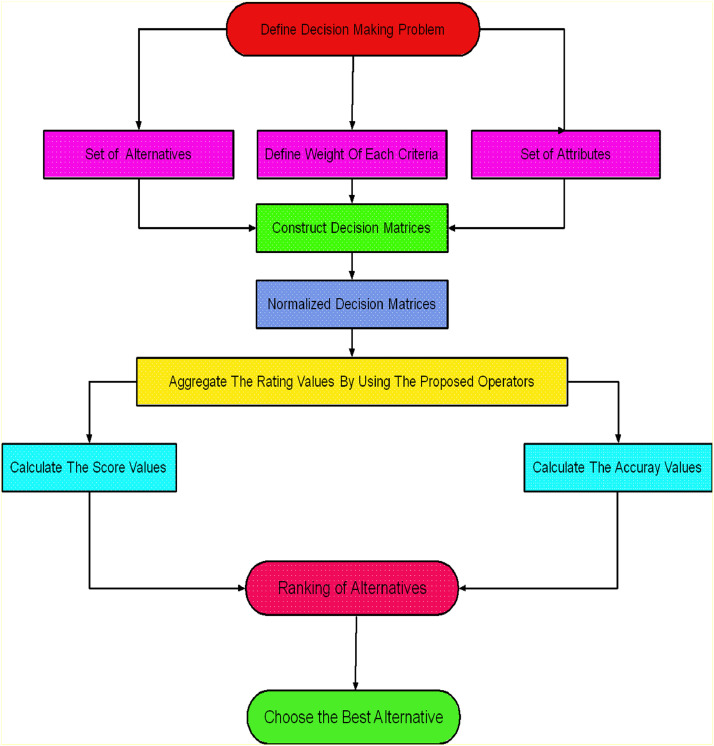
Layout of proposed Model.

In real-world decision-making scenarios, the DM plays a vital role in selecting the most suitable option from a range of alternatives. This section addresses MADM issues using *q*-ROPF$S_{t}$ information through a structured stepwise algorithm and the graphical representation of Algorithm 1 as highlighted in Fig. 6.

**Algorithm 1:** XXX.

Input: Step 1. Establish the decision matrix M${\left[T_{{\ddot{e}}_{ɨʝ}}\right]}_{m\times n\;}.\;$Collect all relevant information for each alternative, expressed in terms of q-ROPF$S_{f}N_{s}\;$and compile it into a comprehensive decision matrix, which captures the degree of tripled membership grade, i.e., positive, neutral, and negative, with each criteria under uncertainty. This provides a more flexible, comprehensive representation of decision-makers' assessment. M=${\left[T_{{\ddot{e}}_{ɨʝ}}\right]}_{m\times n\;}=\left[\begin{array}{cccc} \left(N_{11},M_{11},L_{11}\right) & \left(N_{12},M_{12},L_{12}\right) & \cdots & \left(N_{1m},M_{1m},L_{1m}\right)\\ \left(N_{21},M_{21},L_{21}\right) & \left(N_{22},M_{22},L_{22}\right) & \cdots & \left(N_{2m},M_{2m},L_{2m}\right)\\ \vdots & \vdots & \ddots & \vdots \\ \left(N_{1m},M_{1m},L_{1m}\right) & \left(N_{2m},M_{2m},L_{2m}\right) & \cdots & \left(N_{\mathrm{nm}},M_{\mathrm{nm}},L_{\mathrm{nm}}\right) \end{array}\right].$ Step 2. If all criteria are of the same type, normalization is unnecessary. However, when dealing with different types of criteria, such as profit and cost, the cost-type criteria are converted into profit-type criteria using the normalization formula provided below. $T_{q-\mathrm{ROPF}S_{f}}=\{\begin{array}{c} {\left(T_{q-\mathrm{ROPF}S_{f}}\right)}^{C}\;for\;cost-type\;criteria\\ (T_{q-\mathrm{ROPF}S_{f}}\;)\;for\;benefit\;type\;criteria \end{array}$ Step 3. Employ the proposed AOs to aggregate each alternative. $q-\mathrm{ROPF}S_{f}\mathrm{WG}\left(T_{{\ddot{e}}_{11}},T_{{\ddot{e}}_{12}},\ldots .,T_{{\ddot{e}}_{\mathrm{nm}}}\right)\;=\left(\begin{array}{c} \Pi_{j=1}^{m}{\left(\Pi_{i=1}^{n}N_{\mathrm{ij}}^{\omega_{i}}\right)}^{\upsilon_{j}},\\ {\left(1-\prod_{j=1}^{m}{\left(\prod_{i=1}^{n}{\left(1-M_{\mathrm{ij}}^{q}\right)}^{\omega_{i}}\right)}^{\upsilon_{j}}\right)}^{\frac{1}{q}},\\ {\left(1-\prod_{j=1}^{m}{\left(\prod_{i=1}^{n}{\left(1-L_{\mathrm{ij}}^{q}\right)}^{\omega_{i}}\right)}^{\upsilon_{j}}\right)}^{\frac{1}{q}}\; \end{array}\right)$ Step 4. Assess the score value, using the formula: S$\left(T_{e_{tg}}\right)=\;{\left(\alpha_{tg}\right)}^{q}$−${\left(\beta_{tg}\right)}^{q}-{\left(\delta_{tg}\right)}^{q}$+$\;\left(\frac{e^{{\left(\alpha_{tg}\right)}^{q}-{\left(\beta_{tg}\right)}^{q}-{\left(\delta_{tg}\right)}^{q}}}{e^{{\left(\alpha_{tg}\right)}^{q}-{\left(\beta_{tg}\right)}^{q}-{\left(\delta_{tg}\right)}^{q}}+1}-\frac{1}{2}\right)\pi_{L_{e_{tg}}}^{q},\left(q\;\geq 1\;\right)$. Step 5. To identify the ideal choice, once the values have been ordered. Output: The decision will be based on the alternative with the greatest score.

Fig. 6 express the visual representation of Algorithm 1.

Algorithm 2: CODAS Methodology

**Algorithm 2:** XXX.

Input: Step 1. Taking into account the number of various alternatives and attributes, a decision matrix is built using q − ROPFS_f_Ns. The expert decision matrix is denoted as $\mathrm{EM}=\{EM_{1},EM_{2},\ldots ,EM_{k}\}$ represented the EM matrix. The following represented the kth EM: ${\left[M_{\mathrm{ij}}\right]}_{m\times n}={\left[\begin{array}{cccc} \left(N_{11},M_{11},L_{11}\right) & \left(N_{12},M_{12},L_{12}\right) & \cdots & \left(N_{1m},M_{1m},L_{1m}\right)\\ \left(N_{21},M_{21},L_{21}\right) & \left(N_{22},M_{22},L_{22}\right) & \cdots & \left(N_{2m},M_{2m},L_{2m}\right)\\ \vdots & \vdots & \ddots & \vdots \\ \left(N_{1m},M_{1m},L_{1m}\right) & \left(N_{2m},M_{2m},L_{2m}\right) & \cdots & \left(N_{\mathrm{nm}},M_{\mathrm{nm}},L_{\mathrm{nm}}\right) \end{array}\right]}_{m\times n}.$ Step 2. If all the evaluation criteria are the same, skip the normalization process. However, when the criteria include a mix of types, such as both benefit-type and cost-type, in such a case, normalization becomes necessary, and criteria are transformed into equivalent benefit-type criteria to maintain consistency in the evaluation process. Step 3. To compute the weighted decision matrix. This involves applying the following formula to determine the weighted performance values: $M_{\mathrm{ij}}\;=\left(\begin{array}{c} \Pi_{j=1}^{m}{\left(\Pi_{i=1}^{n}N_{\mathrm{ij}}^{\omega_{i}}\right)}^{\upsilon_{j}},\\ \sqrt[{q}]{1-\prod_{j=1}^{m}{\left(\prod_{i=1}^{n}{\left(1-M_{\mathrm{ij}}^{q}\right)}^{\omega_{i}}\right)}^{\upsilon_{j}}},\\ \sqrt[{q}]{1-\prod_{j=1}^{m}{\left(\prod_{i=1}^{n}{\left(1-L_{\mathrm{ij}}^{q}\right)}^{\omega_{i}}\right)}^{\upsilon_{j}}} \end{array}\right)$(17) Step 4. To compute the negative ideal solution: $\mathrm{NS}={\left[NS_{j}\right]}_{1\times n}$(18) $NS_{j}=\min M_{\mathrm{ij}}$(19) Step 5. Calculate the Euclidean distance and Taxicab distance for the alternatives, using the negative-ideal solution ($NS_{j})\;\mathrm{as}$ $E_{i}=\sqrt{\sum_{j=1}^{n}d_{E}{\left(M_{\mathrm{ij}}-N_{j}\right)}^{2}}$(20) $T_{i}=\sum_{j=1}^{n}d_{T}\left|M_{\mathrm{ij}}-N_{j}\right|$(21) Step 6. Develop the matrix of relative assessments, which is presented as follows: $\mathrm{Rl}={\left[P_{\mathrm{ik}}\right]}_{m\times m}$(22) Where $P_{\mathrm{ik}}=\left(E_{i}-E_{k}\right)+(\psi \left(E_{i}-E_{k}\right)\times (T_{i}-T_{k}$).(23) Where k∈N and ψ is a threshold function that can be used to determine whether two alternatives’ Euclidean distances are equivalent, and are calculated as: $\psi \left(x\right)=\{\begin{array}{c} 1\;if\;|x|\geq \tau \\ 0\;if\;|x|\leq \tau \end{array}$(24) The decision-maker can assign a threshold parameter, represented as τ, which should be in the range of 0.01 to 0.05. If the Euclidean distances between two alternatives differ by less than τ, their comparison is conducted using the taxicab distance. In this study, the value of τ is set as 0.02 for all calculations. Step 7. Calculate the relative assessment score, as follows: $\mathrm{AS}=\sum_{k=1}^{m}P_{\mathrm{ik}}$(25) Step 8. Rank the alternatives according to the decreasing assessment score values.

**Fig 7:**
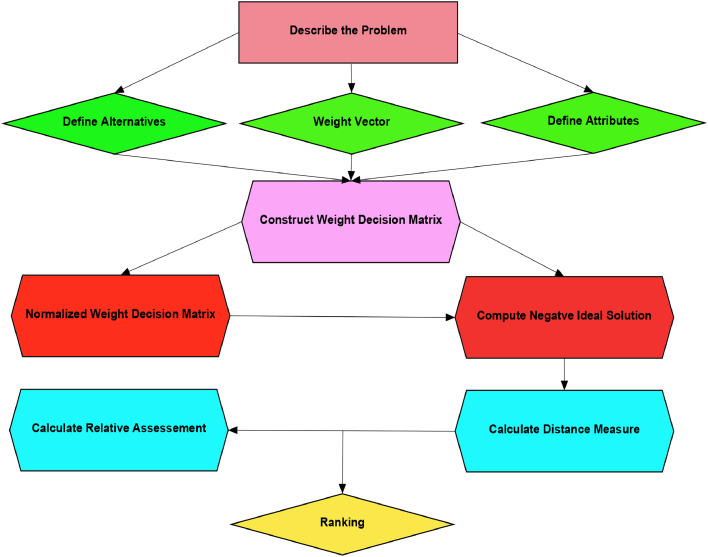
expresses the visual representation of Algorithm 2.

The CODAS method, developed by [42], has proven to be an effective solution for MAGDM techniques. This methodology integrates both Euclidean and taxicab distances to evaluate alternative options. For evaluating alternatives, Euclidean distance serves as the principal evaluation measure, while taxicab distance serves a secondary role. The best solution is identified as the one with the highest distance from the negative-ideal solution [43]. The following outlines the steps of the fuzzy CODAS technique for MAGDM problems and also the graphical representation of Algorithm 2 as highlighted in the decision tree of Fig. 7.

### Application with the help of a numerical example

We demonstrate a MAGDM problem using q − ROPFS_f_Ns with aggregation operators. Two algorithms are developed: the first is a simple aggregation technique, and the second utilizes the CODAS method to solve numerical problems. To demonstrate a numerical case study related to stock investment selection.

**Case Study:**

Organizations often accumulate surplus funds, which can be strategically invested to support growth and reduce risk. Stock selection plays a key role in making informed investment decisions. Companies should prioritize long-term investments over short-term speculation, focusing on fundamentally undervalued stocks with strong growth potential and stability. Long-term value investing encourages evaluating a company’s prospects and avoiding economically sensitive stocks. Given the multiple criteria involved, stock selection is a typical Multi-Attribute Decision-Making (MADM) problem [[42], [44], [45]].

After analysis, we consider five indicators for selecting stocks for investment, which express the set of attributes represented as $n_{j}$(j=1,2,3,4,5) and defined as

$n_{1}$= Industrial Development Prospect.

$n_{2}$= Economic Environment Impact.

$n_{3}$= Sustainable Competitiveness.

$n_{4}$= Market Price vs. Intrinsic Value.

$n_{5}$= Corporate Governance.

The panel of experts has the weight ω=${\left(0.35,0.25,0.21,0.19\right)}^{T}$ Evaluate five alternatives$\;\{L_{1},L_{2},L_{3},L_{4},L_{5}\},$ having WV υ=${\left(0.28,0.20,0.1,0.15,0.27\right)}^{T}$, against the criteria using q-ROPF$S_{t}$ model. The team assessed the five attributes of the investment alternatives, with the evaluation results presented in Tables [Table 1, Table 2, Table 3, Table 4]. Now, use the proposed operator to get the best stock investments in the form of q-ROPF$S_{f}N_{s}$.

**Algorithm-I: Using aggregation methodology**.

**Step 1**. Build a decision matrix M = ${\left[T_{{\ddot{e}}_{ɨʝ}}\right]}_{m\times n\;}$using q-ROPF$S_{t}N_{s}$ provided in Tables [Table 6, Table 7, Table 8, Table 9], respectively

**Table 6:** q-ROPF$S_{t}$ matrix for expert $m_{1}$.

	$n_{1}$	$n_{2}$	$n_{3}$	$n_{4}$	$n_{5}$
$L_{1}$	(.55, .20, .10)	(.55, .33, .11)	(.46, .20, .10)	(.18, .19, .10)	(.65, .22, .10)
$L_{2}$	(.65, .22, .11)	(.80, .10, .10)	(.50, .20, .10)	(.50, .15, .10)	(.40, .10, .10)
$L_{3}$	(.77, .13, .10)	(.60, .25, .15)	(.70, .20, .10)	(.10, .30, .10)	(.50, .23, .11)
$L_{4}$	(.30, .30, .20)	(.40, .13, .10)	(.30, .20, .30)	(.40, .20, .10)	(.22, .30, .20)
$L_{5}$	(.20, .40, .20)	(.35, .30, .10)	(.60, .10, .10)	(.60, .23, .10)	(.30, .30, .20)

**Table 7:** q-ROPF$S_{t}$ matrix for expert $m_{2}$.

	$n_{1}$	$n_{2}$	$n_{3}$	$n_{4}$	$n_{5}$
$L_{1}$	(.55, .30, .10)	(.50, .22, .11)	(.74, .20, .10)	(.40, .30, .10)	(.50, .26, .10)
$L_{2}$	(.20, .14, .20)	(.30, .10, .20)	(.60, .12, .11)	(.22, .17, .10)	(.80, .10, .10)
$L_{3}$	(.72, .11, .10)	(.40, .25, .10)	(.40, .13, .11)	(.50, .22, .20)	(.70, .20, .10)
$L_{4}$	(.6, .27, .12)	(.60, .10, .20)	(.70, .10, .20)	(.63, .13, .11)	(.40, .25, .15)
$L_{5}$	(.66, .23, .11)	(.20, .22, .12)	(.50, .25, .15)	(.70, .17, .10)	(.50, .30, .20)

**Table 8:** q-ROPF$S_{t}$ matrix for expert $m_{3}$.

	$n_{1}$	$n_{2}$	$n_{3}$	$n_{4}$	$n_{5}$
$L_{1}$	(.50, .20, .30)	(.77, .20, .15)	(.88, .22, .11)	(.81, .18, .11)	(.79, .20, .10)
$L_{2}$	(.66, .22, .11)	(.85, .12, .11)	(.70, .30, .15)	(.75, .15, .10)	(.74, .40, .14)
$L_{3}$	(.70, .20, .10)	(.75, .25, .15)	(.84, .12, .11)	(.86, .20, .10)	(.86, .20, .10)
$L_{4}$	(.20, .10, .10)	(.70, .18, .11)	(.75, .25, .10)	(.7, .25, .15)	(.65, .16, .11)
$L_{5}$	(.10, .30, .20)	(.80, .19, .10)	(.74, .20, .10)	(.60, .30, .20)	(.50, .30, .10)

**Table 9:** q-ROPF$S_{t}$ matrix for expert $m_{4}$.

	$n_{1}$	$n_{2}$	$n_{3}$	$n_{4}$	$n_{5}$
$L_{1}$	(.60, .30, .10)	(.50, .22, .10)	(.66, .10, .20)	(.33, .25, .10)	(.30, .30, .10)
$L_{2}$	(.50, .25, .10)	(.30, .20, .10)	(.74, .16, .10)	(.66, .10, .20)	(.71, .10, .17)
$L_{3}$	(.64, .25, .10)	(.40, .20, .10)	(.50, .24, .10)	(.50, .30, .20)	(.60, .26, .12)
$L_{4}$	(.30, .40, .20)	(.60, .10, .30)	(.40, .20, .10)	(.80, .10, .10)	(.55, .25, .15)
$L_{5}$	(.70, .10, .20)	(.70, .20, .10)	(.25, .25, .15)	(.40, .30, .20)	(.40, .30, .10)

**Step 2.** Normalization is not required, due to the uniformity of parameter scales.

**Step 3.** To compute the overall score of each alternative, we apply q-ROPF$S_{t}$WG operator as follow

$$q-\text{ROPF}S_{t}\text{WG}\left(T_{{\ddot{e}}_{11}},T_{{\ddot{e}}_{12}},\ldots .,T_{{\ddot{e}}_{\text{nm}}}\right)\;=\;\left(\begin{array}{c} \Pi_{j=1}^{m}{\left(\Pi_{i=1}^{n}N_{ij}^{\omega_{i}}\right)}^{\upsilon_{j}},\sqrt[{q}]{1-\prod_{j=1}^{m}{\left(\prod_{i=1}^{n}{\left(1-M_{ij}^{q}\right)}^{\omega_{i}}\right)}^{\upsilon_{j}}}\\ ,\\ \sqrt[{q}]{1-\prod_{j=1}^{m}{\left(\prod_{i=1}^{n}{\left(1-L_{ij}^{q}\right)}^{\omega_{i}}\right)}^{\upsilon_{j}}} \end{array}\right)$$

Hence the result is shown in Table 10.

**Table:** 

	n
$L_{1}$	(0.950028, 0.105016, 0.06302)
$L_{2}$	(0.973659, 0.073313, 0.05201)
$L_{3}$	(0.993915, 0.067334, 0.03826)
$L_{4}$	(0.982169, 0.081347, 0.082078)
$L_{5}$	(0.937912, 0.114653, 0.064711)

**Table 10:** Aggregated values of alternative w.r.t. to q-ROPF$S_{t}$WG operator.

	$n_{1}$	$n_{2}$	$n_{3}$	$n_{4}$	$n_{5}$
$L_{1}$	(0.85, 0.17, 0.12)	(0.85, 0.18, 0.08)	(0.9, 0.1, 0.09)	(0.7, 0.16, 0.10)	(0.8, 0.16, 0.07)
$L_{2}$	(0.86, 0.14, 0.09)	(0.88, 0.09, 0.09)	(0.9, 0.1, 0.08)	(0.9, 0.10, 0.10)	(0.9, 0.16, 0.08)
$L_{3}$	(0.97, 0.12, 0.07)	(0.94, 0.16, 0.09)	(0.9, 0.1, 0.07)	(0.9, 0.18, 0.10)	(01, 0.14, 0.07)
$L_{4}$	(0.85,0 .2, 0.11)	(0.91, 0.09, 0.13)	(0.9, 0.1, 0.15)	(0.9, 0.12, 0.10)	(0.9, 0.17, 0.11)
$L_{5}$	(0.72, 0.21, 0.12)	(0.79, 0.16, 0.07)	(0.8, 0.1, 0.08)	(0.9, 0.17, 0.10)	(0.8, 0.19, 0.11)

**Step 4.** In this step, we compute the score values of each alternative, by using the score function define as

S$\left(T_{e_{tg}}\right)=\;{\left(\alpha_{tg}\right)}^{q}$−${\left(\beta_{tg}\right)}^{q}-{\left(\delta_{tg}\right)}^{q}$+$\;\left(\frac{e^{{\left(\alpha_{tg}\right)}^{q}-{\left(\beta_{tg}\right)}^{q}-{\left(\delta_{tg}\right)}^{q}}}{e^{{\left(\alpha_{tg}\right)}^{q}-{\left(\beta_{tg}\right)}^{q}-{\left(\delta_{tg}\right)}^{q}}+1}-\frac{1}{2}\right)\pi_{L_{e_{tg}}}^{q},\left(q\;\geq 1\;\right)$, as shown in Table 11.

**Table 11:** Score values of the alternative.

Score Values	q-ROPF$S_{t}$WG
$L_{1}$	0.961196
$L_{2}$	0.922504
$L_{3}$	0.981495
$L_{4}$	0.946365
$L_{5}$	0.823284

**Step 5**. After ordering the values, the most suitable option is identified.

$$L_{3}>L_{1}>L_{4}>L_{2}>L_{5}$$

**Fig. 8:**
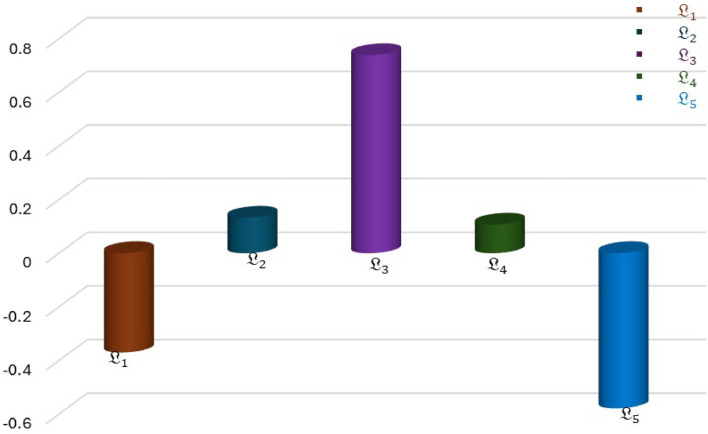
Expressed the ranking order of q-ROPF$S_{t}$WG operator.

The analysis indicates that $L_{3}$ the most suitable option among all the considered options. The ranking order of alternatives is expressed in the Fig. 8.

### Algorithm-II: using CODAS methodology

**Step 1.** Expert information in the form of q-ROPF$S_{t}N_{s}$ is shown in Tables 5-8, with five alternatives

$L_{i}\left(i=1,2\ldots ,5\right)$ and five attributes $n_{j}\left(j=1,2,\ldots 5\right)$ and $m_{k}$(k=1,2,3,4) represented the expert matrices.

**Step 2.** Since each parameter is assessed on an identical basis, which normalization of the expert matrices can be omitted.

**Step 3.** The weighted normalized expert matrix was calculated by using the equation as define as:

$$M_{\mathrm{ij}}\;=\Pi_{j=1}^{m}{\left(\Pi_{i=1}^{n}N_{\mathrm{ij}}^{\omega_{i}}\right)}^{\upsilon_{j}},\sqrt[{q}]{1-\prod_{j=1}^{m}{\left(\prod_{i=1}^{n}{\left(1-M_{\mathrm{ij}}^{q}\right)}^{\omega_{i}}\right)}^{\upsilon_{j}}},\sqrt[{q}]{1-\prod_{j=1}^{m}{\left(\prod_{i=1}^{n}{\left(1-L_{\mathrm{ij}}^{q}\right)}^{\omega_{i}}\right)}^{\upsilon_{j}}}$$

So we get the results are shown in Table 12.

**Table 12:** Weighted normalized matrix by q-ROPF$S_{t}\text{WG}$ operator.

	$n_{1}$	$n_{2}$	$n_{3}$	$n_{4}$	$n_{5}$
$L_{1}$	(0.85, 0.17, 0.12)	(0.85, 0.18, 0.08)	(0.9, 0.1, 0.09)	(0.7, 0.16, 0.10)	(0.8, 0.16, 0.07)
$L_{2}$	(0.86, 0.14, 0.09)	(0.88, 0.09, 0.09)	(0.9, 0.1, 0.08)	(0.9, 0.10, 0.10)	(0.9, 0.16, 0.08)
$L_{3}$	(0.97, 0.12, 0.07)	(0.94, 0.16, 0.09)	(0.9, 0.1, 0.07)	(0.9, 0.18, 0.10)	(01, 0.14, 0.07)
$L_{4}$	(0.85,0 .2, 0.11)	(0.91, 0.09, 0.13)	(0.9, 0.1, 0.15)	(0.9, 0.12, 0.10)	(0.9, 0.17, 0.11)
$L_{5}$	(0.72, 0.21, 0.12)	(0.79, 0.16, 0.07)	(0.8, 0.1, 0.08)	(0.9, 0.17, 0.10)	(0.8, 0.19, 0.11)

**Step 4.** Now using a weighted geometric operator for Ψ = 2. To get negative ideal solution in the form of q-ROPF$S_{t}N_{s}$ as shown in Table 13.

**Table 13:** Negative ideal solution by q-ROPF$S_{t}\text{WG}$ operator.

	$n_{1}$	$n_{2}$	$n_{3}$	$n_{4}$	$n_{5}$
L	(0.72, 0.21, 0.12)	(0.79, 0.18, 0.13)	(0.8, 0.1, 0.15)	(0.7, 0.18, 0.10)	(0.8, 0.19, 0.11)

**Step 5.** From negative ideal solution ($NS_{j})\;$as shown in Table 13, we compute Euclidean distance $E_{i}=\sqrt{\sum_{j=1}^{n}d_{E}{\left(M_{ij}-N_{j}\right)}^{2}}$ and Taxicab distance $T_{i}=\sum_{j=1}^{n}d_{T}\left|M_{ij}-N_{j}\right|$, for the alternatives as shown in Table 14.

**Table 14:** Distance of q-ROPF$S_{t}\text{WG}$ operator.

	Euclidean Distance	Taxicab Distance
$L_{1}$	0.2	0.16
$L_{2}$	0.3	1
$L_{3}$	0.42	1.24
$L_{4}$	0.3	0.8
$L_{5}$	0.16	0.29

**Step 6.** Table 15. Provides the relative assessment matrix for q-ROPF$S_{t}\text{WG}$ operator.

**Table 15:** Relative assessment of q-ROPF$S_{t}\text{WG}$.

	$n_{1}$	$n_{2}$	$n_{3}$	$n_{4}$	$n_{5}$
$L_{1}$	0	-0.1	-0.2	-0.1	0
$L_{2}$	0.1	0	-0.1	0	0.1
$L_{3}$	0.22	0.12	0	0.1	0.3
$L_{4}$	0.1	-0	-0.1	0	0.1
$L_{5}$	-0	-0.1	-0.3	-0.1	0

**Step 7.** Calculate the relative assessment score using the formula: $AS=\sum_{k=1}^{m}P_{ɨk}$. Hence the result is shown in Table 16.

**Table 16:** Assessment score.

Alternative	Score Values
$L_{1}$	-0.4
$L_{2}$	0.13
$L_{3}$	0.74
$L_{4}$	0.11
$L_{5}$	-0.6

**Step 8.** At the end to get the best option, first we rank the alternatives according to the decreasing assessment score values.

$L_{3}>L_{2}>L_{4}>L_{1}>L_{5}$.

The evaluation metrics can be considered from different perspectives to validate the proposed q-rung orthopair picture fuzzy soft based framework with CODAS method. Consistency can be checked by observing whether small variations in expert opinions or input data lead to stable rankings of alternatives. Comparative validity can be examined by comparing the obtained rankings with results from other well-established MADM approaches to assess alignment. Robustness can be measured by testing the framework under different weighting schemes or parameter values of q, ensuring that the decision outcomes remain reasonable. Furthermore, computational efficiency can be evaluated by analyzing the time complexity of the aggregation and CODAS steps, while interpretability serves as a practical metric, highlighting whether the decision process and final rankings are understandable for decision-makers. These metrics collectively ensure that the proposed method is not only mathematically rigorous but also practically reliable for real-world investment decision-making.

### Managerial implications

In this section, the managerial implication of the generalized structure of q−rung orthopair picture fuzzy soft sets helps decision-makers make strong and reliable decisions. To check the stability of the proposed model, we performed various analysis tests to check the stability, authenticity, superiority, and influence of our proposed model**.**

### Validity test

**Fig. 9:**
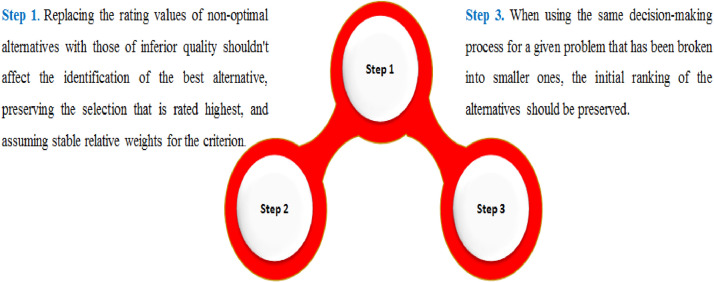
Steps of the validity test.

To illustrate the adaptability of the proposed technique in various settings, this study utilizes the evaluation protocols developed by Wang and Trianaphyllou [46] in the following ways in Fig. 9.

**Test of validity employing criteria 1.**

Using the proposed method, the ranking of alternatives is $L_{3}>L_{2}>L_{4}>L_{1}>L_{5}$.

Based on test criteria 1, we replaced the non-optimal alternative. $L_{1}$ with the lowest alternative $L_{1}^{*}$. To evaluate the stability of the proposed method.

(0.3927,0.2749,0.1487),(0.4723,0.2180,0.1474),(0.5810,0.2470,0.1499) and (0.5019,0.2492,0.1610), (0.4711,0.2073,0.1320) were used as the rating values of $L_{5}^{*}.\;$Using the suggested methodology aggregates the alternatives by using the score values: $S(L_{1})$ = 0.0448, $S(L_{2})$= 0.1120, $S(L_{3})$=0.2125 and $S(L_{4})$= 0.1296, $S(L_{5})$= -0.1047, so the result of the ranking order is $L_{3}>L_{2}>L_{4}>L_{1}>L_{5}^{*}$. With the best alternative remaining the same as the suggested approach. Thus, the findings consistently support test criteria 1.

**Test of validity employing criteria 2 and 3**.

The fragmented decision-making subcases are regarded as $\left\{L_{2},L_{1},L_{3}\right\}$, $\left\{L_{4},L_{1},L_{2}\right\}$ and $\left\{L_{3},L_{2},L_{4}\right\}$ to assess the validity based on criteria 2 and 3. They rank in the following sequence via the procedures mentioned: $L_{2}>L_{1}>L_{3}$, $L_{4}>L_{1}>L_{5}$ and $L_{3}>L_{2}>L_{5}$. After combining all the findings, the overall ranking appears as $L_{3}>L_{2}>L_{4}>L_{1}>L_{5}$, which is exactly in line with the outcomes of the initial decision-making process. As a result, our suggested strategy meets requirements 2 and 3.

### Sensitivity analysis

**Fig. 10:**
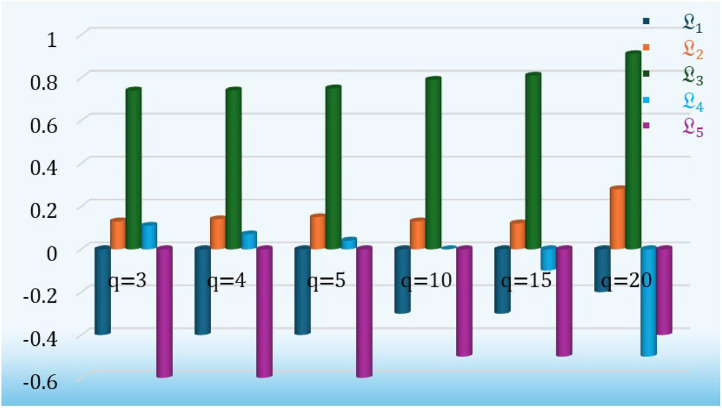
The behavior of score values concerning q, by using q-ROPF$S_{t}$WG operator.

In this section, we performed a parameter analysis test to evaluate the stability and influence of our proposed model under varying values of parameter q = (3,4,5,10,15, and 20) by using the proposed aggregation operators. For this analysis, we employ q-ROPF$S_{t}$WG operator under the various values of q = 3,4,5,10,15, and 20 as shown in Table 17 and Fig. 10.

**Table 17:** Score values of the alternative by q-ROPF$S_{t}$WG operator under various values of q.

q	Score values					Ranking	Best alternative
	$L_{1}$	$L_{2}$	$L_{3}$	$L_{4}$	$L_{5}$		
3	-0.4	0.13	0.74	0.11	-0.6	$L_{3}>L_{2}>L_{4}>L_{1}>L_{5}$	$L_{3}$
4	-0.4	0.14	0.74	0.07	-0.6	$L_{3}>L_{2}>L_{4}>L_{1}>L_{5}$	$L_{3}$
5	-0.4	0.15	0.75	0.04	-0.6	$L_{3}>L_{2}>L_{4}>L_{1}>L_{5}$	$L_{3}$
10	-0.3	0.13	0.79	0.0	-0.5	$L_{3}>L_{2}>L_{4}>L_{1}>L_{5}$	$L_{3}$
15	-0.3	0.12	0.81	-0.1	-0.5	$L_{3}>L_{2}>L_{4}>L_{1}>L_{5}$	$L_{3}$
20	-0.2	0.28	0.91	-0.5	-0.4	$L_{3}>L_{2}>L_{1}>L_{5}>L_{4}$	$L_{3}$

After examining the data in Table 16, we indicate that while the score values of q-ROPF$S_{t}$WG operator decreases while increasing the values of “q”, the top-ranked alternative (L₃) remains consistent in most cases. This demonstrates the robustness and stability of the proposed model, even under fluctuating parameter values. However, we also observed that at significantly higher values of “q”, such as q=20, minor deviations in the ranking order occur. This suggests that extremely large values of q may introduce unnecessary sensitivity. Therefore, for practical applications, we recommend selecting moderate values of q**,** such as 5 or 10**,** which offer a balanced trade-off between flexibility and ranking stability**.** Despite this, the analysis demonstrates that the proposed approach maintains robust decision-making stability, enabling reliable, educated judgments based on the result.

### Comparative analysis

**Fig. 11:**
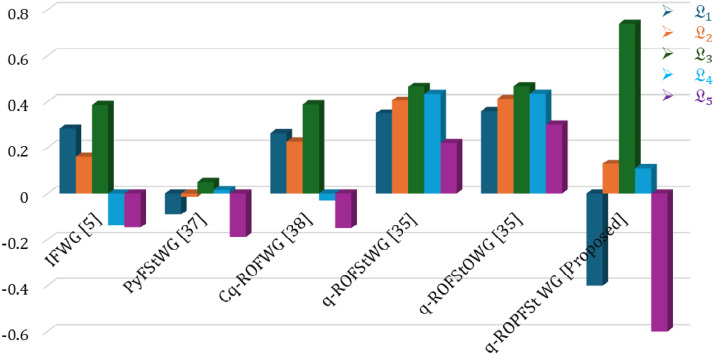
Expressed the comparative analysis of various operators.

In this section, our main concern is to demonstrate the authenticity and superiority of our proposed model. For this purpose, we perform a comparison analysis of the proposed model with existing approaches such as [5], [37],[38] and[35] to utilize a variety of proposed operators. Our primary goal in this section is to compare the ranking order of alternatives generated by the proposed model with existing approaches. Significantly, the consistency of the ranking order of alternatives, with the same best alternative, underscores the flexibility and comprehensiveness of our proposed technique. As compared to existing approaches, our proposed model demonstrates enhanced flexibility and liberated structure, empowering decision-makers to navigate a decision-making process more realistically. This flexibility is given in Table 18 and Fig. 11.

**Table 18:** Comparison with various existing approaches.

Aggregation Operators	Score values					Best Alternative
	$L_{1}$	$L_{2}$	$L_{3}$	$L_{4}$	$L_{5}$	
IFWG [5]	0.2819	0.1607	0.3850	-0.1365	-0.1471	$L_{3}$
$P_{y}FS_{t}\mathrm{WG}$ [36]	-0.0911	-0.0132	0.0505	0.0159	-0.1877	$L_{3}$
Cq-ROFWG [47]	0.2630	0.2258	0.3879	-0.0317	-0.1507	$L_{3}$
q-ROF$S_{t}\mathrm{WG}$ [35]	0.3493	0.4048	0.4648	0.4337	0.2197	$L_{3}$
q-ROF$S_{t}\mathrm{OWG}$[35]	0.3600	0.4132	0.4677	0.4342	0.3012	$L_{3}$
q-ROPF$S_{t}\mathrm{WG}$ [Proposed]	-0.4	0.13	0.74	0.11	-0.6	$L_{3}$

### Characteristic analysis

In this section, we show the characteristic analysis of the proposed structure with existing theories cited in [2], [10], [18], [35] and [28] are c. Upon examining Table 19, the existing model overlooks neutral degree and does not utilize parameterization tools, which are essential for representing real-world complexities. The unique distinguishing characteristic of the proposed model is its capability to effectively handle real-life decision-making scenarios by positive, neutral, and negative degrees, along with flexible parameterization mechanisms. While existing methods have certain limitations within a constrained domain. To address these shortcomings, the proposed structure introduced a more adaptable environment through a q-rung orthopair picture fuzzy soft framework, governed by the condition 0 ≤ $\mu^{q}+\eta^{q}+\xi^{q}\leq 1.$ Consequently, our model provides a more robust and realistic decision-making foundation compared to traditional approaches.

**Table 19:** Characteristic analysis of various theories.

Methods	Mem	Neutral	Non-mem	Parameter	Domain
IFS [2]	✓	✕	✓	✕	0≤μ+η≤1
PyFS [10]	✓	✕	✓	✕	$0\leq \mu^{2}+\eta^{2}\leq 1$
q-ROFS [18]	✓	✕	✓	✕	$0\leq \mu^{q}+\eta^{q}\leq 1\;\left(q\geq 1\right)$
q-ROF$S_{t}S$ [35]	✓	✕	✓	✓	$0\leq \mu^{q}+\eta^{q}\leq 1\;\left(q\geq 1\right)$
PFS [28]	✓	✓	✓	✕	0≤μ+η+ξ≤1
[This Paper]	✓	✓	✓	✓	$0\leq \mu^{q}+\eta^{q}+\xi^{q}\leq 1\;\left(q\geq 1\right))$

**Fig. 12:**
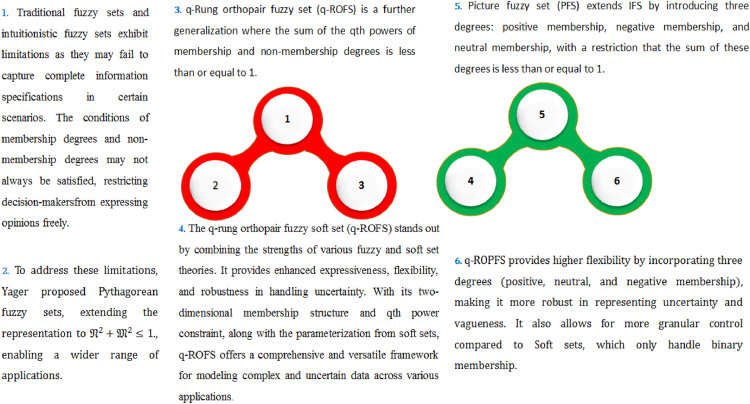
Advantages of q-SFR sets compared to already existing structures.

Based on several comparative analyses, we emphasize the benefit of using the notion of q-rung orthopair picture fuzzy soft sets, which are visually represented in Fig. 12.

### Novelty and theoretical improvements

To clarify the novelty of our proposed q-ROPF$S_{t}\text{WG}$ operator, we highlight both its mathematical generalization and practical enhancement over existing operators such as $P_{y}FS_{t}\text{WG}\left[37\right]$ and q-ROF$S_{t}\text{WG}$ [35] having certain limitations within a constrained domain and also handling uncertainty concerning membership and non-membership functions, having no information about the neutral degree, which are essential for representing real-world complexities. To address these shortcomings, the proposed structure introduced a more adaptable environment through a q-rung orthopair picture fuzzy soft framework, governed by the condition 0 ≤ $\mu^{q}+\eta^{q}+\xi^{q}\leq 1$. The unique distinguishing characteristic of the proposed model is its capability to effectively handle real-life decision-making scenarios by positive, neutral, and negative degrees, along with flexible parameterization mechanisms. This broader framework captures uncertainty more accurately, particularly in real-world MCDM problems. Furthermore, the incorporation of soft set theory in our operator brings parameter-based adaptability, making the model capable of handling decision-maker preferences more realistically. Additionally, the new operator maintains all essential mathematical properties such as idempotency, boundedness, and monotonicity, while offering enhanced flexibility in aggregation via parameters and neutral handling, which are mathematically absent in the previously existing methods. This improvement is validated not only through comparative ranking (Table 17) but also through structural and functional comparisons (Table 18), affirming that our operator provides a more robust, expressive, and realistic decision-support framework.

### Advantages

The proposed operators have the following advantages:

1. The proposed model effectively addresses gaps left by existing theories, particularly in handling neutral degrees and parameterization tools. By integrating these often-overlooked aspects, it provides a more holistic approach to decision-making, enhancing its applicability across various scenarios.
2. The model's unique ability to utilize positive, neutral, and negative membership degrees, combined with parameterization tools, allows it to tackle a wide range of real-world problems. This versatility makes it more effective than traditional approaches in solving complex issues.
3. The model is proficient in handling Multi-Attribute Decision-Making (MADM) problems, especially in situations where decision-making becomes more realistic and complex. Its robustness ensures reliable outcomes even in challenging scenarios.

### Limitations

The proposed operators have the following limitations:

1. While the model demonstrates flexibility and reliability through a case study on profitable returns in mineral resource exploration, its validation is limited to this specific context. To ensure broader applicability, it is essential to test the model across a variety of real-life decision-making challenges in different industries. Expanding the validation scope would provide a more comprehensive understanding of the model's robustness and versatility.
2. The impact of varying the parameter "q" on decision-making outcomes has not been fully explored. This lack of sensitivity analysis may limit the model's reliability in diverse scenarios. Conducting a thorough investigation into how different values of "q" influence the results would provide clearer guidelines and enhance the model's stability and applicability in varying contexts.
3. The model's effectiveness may be constrained by the assumptions underlying its framework, particularly in more complex or unconventional decision-making scenarios. These assumptions, while necessary for tractability, might not align perfectly with real-world conditions. Future research should focus on validating the model in diverse and complex environments to ensure its practical relevance and accuracy.

## Conclusion

This article aims to initiate a joint concept of PFS and q-ROF$S_{t}$S, to get a novel concept of q-ROPF$S_{t}$S, which tackles inherent vagueness in information by applying a triplet of membership linked to specific attributes. In the previous literature theories, there was a lack of information about a neutral degree, and their constraints are restricted by certain limitations. So, to handle these issues, we provide a more flexible environment to the decision maker by relaxing the domain by using q-rung orthopair picture fuzzy soft environment under the condition $0\;\leq \;\mu^{q}+\eta^{q}+\xi^{q}\leq 1\;\left(q\geq 1\right).$ We originate geometric aggregation operators under the proposed environment, such as $q-\text{ROPF}S_{t}\text{WG}$ and $q-\text{ROPF}S_{t}\text{OWG}$ operators, and based on these operators, we define some dominant properties. To solve the MADM issue, we provide a stepwise algorithm to construct an example related to stock investment selection and develop a stepwise algorithm for multi-attribute decision-making problems under the proposed structure and CODAS method. In the end, we make various analysis tests such as parameter analysis test by using the various values of “q”, to check the stability and influence, comparative analysis with an existing method to show the authenticity and characteristic analysis to the superiority of the proposed structure invest their resource in exploration projects related to mineral resources to get profitable return. Hence, our proposed structure is superior and authentic than existing approaches. In the future, we will extend this idea into hyper-soft sets, neutrosophic sets, and various aggregations [[48], [49], [50], [51], [52]].

## Ethics statements

Not applicable

## CRediT author statement

**Sumbal Ali:** Methodology, Writing-review & editing. **Ikram Ullah:** Conceptualization, Methodology, Supervision. **Salma Khan:** Writing-original draft. **Hasib Khan:** Investigation. Hasib Khan: Investigation, Validation. **Hisham Muhammad Alkhawar:** Methodology, Resources. Hisham **Jehad Alzabut:** Investigation, Validation.

## Supplementary material *and/or* additional information [OPTIONAL]

None.

## Declaration of competing interests

The authors declare that they have no known competing financial interests or personal relationships that could have appeared to influence the work reported in this paper.

## Data Availability

Data will be made available on request.

## Appendix Supplementary materials

Image, application 1
